# Statistical Use of Argonaute Expression and RISC Assembly in microRNA Target Identification

**DOI:** 10.1371/journal.pcbi.1000516

**Published:** 2009-09-25

**Authors:** Stephen A. Stanhope, Srikumar Sengupta, Johan den Boon, Paul Ahlquist, Michael A. Newton

**Affiliations:** 1 Department of Statistics, University of Wisconsin–Madison, Madison, Wisconsin, United States of America; 2 Department of Biostatistics and Medical Informatics, University of Wisconsin–Madison, Madison, Wisconsin, United States of America; 3 Biological Sciences Division, University of Chicago, Chicago, Illinois, United States of America; 4 The Wicell Research Institute, Madison, Wisconsin, United States of America; 5 McArdle Laboratory for Cancer Research, University of Wisconsin–Madison, Madison, Wisconsin, United States of America; 6 Institute for Molecular Virology, University of Wisconsin–Madison, Madison, Wisconsin, United States of America; 7 Howard Hughes Medical Institute, University of Wisconsin–Madison, Madison, Wisconsin, United States of America; Washington University in Saint Louis, United States of America

## Abstract

MicroRNAs (miRNAs) posttranscriptionally regulate targeted messenger RNAs (mRNAs) by inducing cleavage or otherwise repressing their translation. We address the problem of detecting m/miRNA targeting relationships in *homo sapiens* from microarray data by developing statistical models that are motivated by the biological mechanisms used by miRNAs. The focus of our modeling is the construction, activity, and mediation of RNA-induced silencing complexes (RISCs) competent for targeted mRNA cleavage. We demonstrate that regression models accommodating RISC abundance and controlling for other mediating factors fit the expression profiles of known target pairs substantially better than models based on m/miRNA expressions alone, and lead to verifications of computational target pair predictions that are more sensitive than those based on marginal expression levels. Because our models are fully independent of exogenous results from sequence-based computational methods, they are appropriate for use as either a primary or secondary source of information regarding m/miRNA target pair relationships, especially in conjunction with high-throughput expression studies.

## Author Summary

MicroRNAs are a family of small RNAs that play important roles in the development, physiological function and stress responses of a wide variety of organisms, and if abnormally expressed are associated with multiple types of cancer in humans. Rather than being translated into proteins, members of the family of microRNAs operate by preventing the translation of messenger RNAs to which they have some degree of sequence complementarity. Although sequence-based bioinformatics techniques have yielded large numbers of predicted messenger- and microRNA targeting relationships, verifying these as *bona fide* has proven practically difficult. We have developed a novel statistical approach based on the system biology of microRNAs in humans to detect such targeting relationships using high-throughput RNA expression data. Because our approach is not based on information from external target pair predictions, it can play a fully independent role in verifying such predictions as well as be used to obtain *de novo* target pair predictions. Using two separate data studies, we show that our approach is capable of both reproducing previously observed target pairs and verifying putative target pairs predicted from sequence data, at rates substantially better than marginal comparisons of messenger- and microRNA expression levels.

## Introduction

Micro RNAs (miRNAs) are small (20–22 bp) RNAs transcribed by a wide variety of organisms, from viruses [1], to plants [2],[3], to animals such as *C. elegans*, *Drosophila* and humans [4]–[6]. While most RNAs function in ribosomes or splicesomes, or are translated into proteins necessary for cellular function, miRNAs instead serve as negative regulators of gene expression by preventing the translation of messenger RNAs (mRNAs). Through their regulatory activities, miRNAs have been shown to affect organismal development, physiological function and stress responses. Abnormal miRNA production has also been associated with the development of several types of cancer [7]–[10].

**Figure 1:**
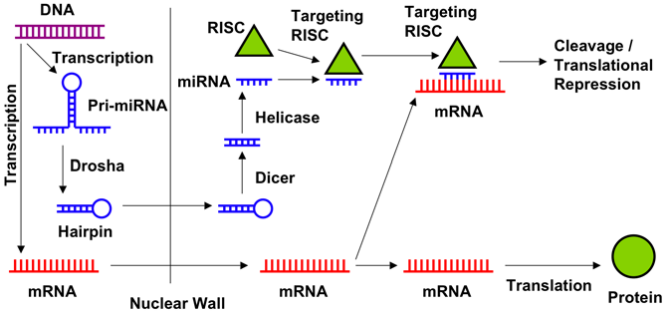
miRNA biogenesis and gene silencing. miRNA biogenesis begins with transcription of a pri-miRNA which is processed into a hairpin, exported from the nucleus, processed into a mature miRNA, and incorporated into a RISC. Minimal functional RISCs consist of an Argonaute protein and a miRNA. RISCs hybridize to mRNAs at targeting sites complementary with the miRNA, and prevent translation by either cleaving the mRNA or maintaining its position at the target site and stopping ribosomal activity.

Posttranscriptional gene silencing through miRNA activity occurs through a multistep process (Figure 1) [11]–[17] with an overall structure that has been remarkably conserved across organisms. This process begins with primary miRNA transcripts (pri-miRNAs) being either transcribed from “miRNA genes” or spliced from the intronic regions of mRNAs. In the nucleus, pri-miRNAs fold into hairpin structures from which trailing 3′ and 5′ ends are cleaved away by the RNase Drosha. The resulting precursors to mature miRNAs (pre-miRNAs) are then exported from the nucleus to the cytoplasm, where a second RNase enzyme (Dicer) removes the hairpin loop. This produces a segment of double stranded RNA that is separated into two single strands by helicase enzymes. After separation, one of the single stranded RNAs is combined with an Argonaute (Ago) protein to form an RNA-induced silencing complex (RISC). (Although other proteins may be incorporated into the structure, an Ago protein and miRNA compose a minimal functional RISC [18],[19].) Once assembled, RISCs composed of a given miRNA interfere with the translation of select mRNAs by hybridizing to them at target sites complementary to the miRNA sequence and either cleaving the mRNA or blocking its translation while leaving the molecule intact. Any mRNA translationally regulated by a particular miRNA can be anticipated to have a limited number of target sites usable by that miRNA. Each miRNA can target multiple mRNAs, and an mRNA may contain target sites for multiple miRNAs.

While both mRNA cleavage and blocking ribosomal activity disrupt translation, the latter does not directly alter mRNA abundance. Whether a particular RISC cleaves or blocks translation is determined by both the qualities of the hybridization and properties of the Ago protein contained in the RISC. The number and function of distinct Ago proteins shows substantial variability across organisms. For example, in *Arabidopsis* there are 10 different variants of Ago and miRNAs preferentially associate with only one in forming RISCs [20],[21], while in humans there are 4 commonly coexpressed Ago proteins, and miRNAs can be effectively regarded to have equal propensity to combine with each [22],[23]. The variant of Ago primarily utilized by miRNAs in *Arabidopsis* is competent for target cleavage [21], which is consistent with previous observations that the dominant means of miRNA-based regulation of mRNA translation is cleavage rather than translational repression. In humans, only RISCs composed of Ago 2 have been demonstrated to have the ability to cleave and degrade targeted mRNAs [22],[24]. Since miRNAs have equal propensity to combine with each of these, it is reasonable to conclude that targeted mRNAs are repressed through a combination of both cleavage and ribosomal blockage. This is consistent with results described by Nakamoto *et al* [25] which demonstrate simultaneous increases in both target mRNA and polyribosomal fraction in human miRNA knockdown studies, and recent experiments reported by Bartel *et al* [26] that suggest in mice (which share many of the complexities found in human Ago properties and RISC formation), most mRNA targets of miRNA-mediated repression are cleaved.

To determine whether a miRNA targets a particular mRNA, sequence-based computational target prediction methods may be used to identify potential miRNA hybridization sites within that mRNA [27]–[34]. Algorithms such as miRanda [35] use m/miRNA alignments and hybridization energies as metrics to score mRNA subsequences, and report high-scoring subsequences as putative target sites. More recently proposed methods additionally utilize evolutionary conservation of a predicted site across multiple organisms (PicTar [36]), information regarding target site position and base content (TargetScan [37]–[39]), or mRNA secondary structure [40] to improve prediction performance. Although existing computational target prediction algorithms provide important information regarding potential m/miRNA target pairings, they are acknowledged to have issues with specificity and sensitivity [29],[30] as well as inter-algorithm consistency [30],[34]. (These issues are discussed in relation to this study in the Methods and Discussion sections.) The problem of how to reliably predict target pair relationships from sequence data alone is currently unresolved.

With the limitations of purely sequence-based methods of miRNA target prediction, it has been suggested that the statistical analysis of expression data may play an important role not only in verifying computationally predicted m/miRNA targeting relationships, but also for generating *de novo* target pair predictions [27],[29]. Such analysis would require that both mRNA and miRNA abundance be measured on the same tissue samples, and naturally would consider the marginal correlation between a miRNA and its putative target. Marginal approaches are attractive because they are simple and they aim to capture the fundamental negative relationship between miRNAs and their targets. However, determining reliable and replicable targeting relationships through marginal expression comparisons either on their own or in combination with computational prediction has proven to be difficult both previously [41] and in our own analysis (see following results).

We hypothesize that statistical models guided by knowledge of the miRNA pathway can be used to reduce error in both validating and predicting targeting relationships. The premise of our approach is that although a negative abundance relationship may exist in an m/miRNA pair, this relationship may only be detectable within the context of the abundance of other molecules that participate in mRNA silencing. In a marginal comparison of m/miRNA expression levels for the purpose of verifying a predicted targeting relationship, miRNA expressions are compared directly to those of a putatively targeted mRNA. When expression data from *homo sapiens* are under study, such a comparison uses miRNA expressions as a direct substitute for those of RISC composed of Ago 2 protein and a targeting miRNA. Additionally, marginal comparisons do not compensate for indirect effects on mRNA abundance caused by the blocking RISCs composed of Ago 1, 3 or 4 proteins and the targeting miRNA. Although *ceteris parabis* increases of the levels of these RISCs cannot observably reduce the concentration of the targeted mRNA, because they utilize the same target sites as RISCs containing Ago 2 such increases can be anticipated to affect the ability of Ago 2 RISCs to cleave targeted mRNAs. Finally, marginal comparisons do not compensate for either the targeting of the mRNA in a putative target pair by RISCs constructed from miRNAs other than that under consideration, or targeting of mRNAs other than the one under analysis by the miRNA.

In this paper, we develop a linear regression model that accounts for a variety of elements and interactions in the human miRNA pathway and that compensates for idiosyncratic aspects of two data collections on which it is applied. Central to this model is the comparison of the expression levels of a putatively targeted mRNA to a proxy for RISC expression composed of an interaction between Ago 2 and a targeting miRNA, rather than to miRNA expression alone. To demonstrate that our approach offers superior performance to marginal m/miRNA comparisons, we compare the two methods on sets of m/miRNA pairs both previously shown and predicted to have targeting relationships using expression data from two different studies as well as a combination of the data. We find that: 1) the system biological regression approach explains a higher proportion of the observed variation in known mRNA target levels, even after compensating for increases in model complexity. 2) The estimated effects of proxies to targeting Ago 2 RISC expressions on the expressions of known mRNA targets are more consistently and appropriately negative than those of marginal miRNA expressions. 3) A larger number of known m/miRNA target pairs are identified as such using the regression approach compared to marginal m/miRNA methods. 4) The system biological regression approach provides evidence supporting substantially more computationally predicted m/miRNA pairs as *bona fide* than do marginal m/miRNA comparisons. Because we obtain these improvements in performance without directly utilizing exogenous information from sequence-based computational target prediction methods, our approach provides a basis for statistical methods to putative m/miRNA target pair analysis that can play useful roles in both verifying computational target predictions as well as generating *de novo* information regarding m/miRNA target relationships.

## Methods

### Regression modeling

There are two categories of covariates that ought to be compensated for when comparing the expression levels from a putative m/miRNA target pair in *homo sapiens* for the purpose of inferring a targeting relationship: those corresponding to elements of the miRNA system biology, and those corresponding to idiosyncratic data effects (if any). Of these two categories, covariates related to the miRNA system biology can be further subdivided into those pertaining to the effect of the particular miRNA under analysis on the putatively targeted mRNA rather than that of other miRNAs potentially targeting the mRNA, those related to observable target cleavage rather than those resulting in translational repression without cleavage through a maintained hybridization at a target site, and those related to the affinity of both the miRNA under analysis as well as other miRNAs to mRNAs not under direct consideration.

It can be presumed that the covariates in these categories are related to one another and to target mRNA expression in a complicated and nonlinear manner, and any statistical or computational procedure for inferring m/miRNA targeting relationships ought to have some degree of fidelity to the system biology represented by the model it is explicitly or implicitly based upon. However, the fidelity of the model also should be balanced against the need for a computationally efficient procedure that works well given the limitations of sample size and the levels of variation in the system. A well-formulated regression model is computationally tractable (especially if large numbers of putative m/miRNA pairs are to be evaluated) and is a standard approach to decomposing variation in a response. Further, although a linear formulation may not emerge from first principles, it may capture the dominant relationships sufficiently well to identify *bona fide* targeting relationships. Thus we relate the categories of system biologic covariates to the expression of a putatively targeted mRNA as in (1):
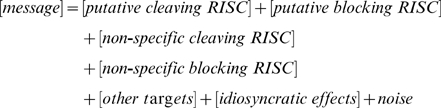
(1)where [*message*] refers to expression of the putative targeted mRNA; [*putative cleaving RISC*] represents the effect of RISCs composed of the putative targeting miRNA and Ago 2 on the targeted mRNA; [*putative blocking RISC*] is the effect of RISCs composed of the putative targeting miRNA and Ago 1, 3 or 4; [*non-specific cleaving RISC*] is the effect of RISCs composed of Ago 2 and miRNAs not under particular consideration; [*non-specific blocking RISC*] is the effect of RISC composed of Ago 1, 3 or 4 and the unconsidered miRNAs; [*other targets*] refers to the effect that the expression of other mRNAs have on the putative targeted mRNA, especially through their affinity for interactions with the putative targeting miRNA; [*idiosyncratic effects*] are dataset-specific effects; *noise* represents natural variation in [*message*] as well as that due to systemic effects not adequately captured in our model.

Although the levels of RISCs of various types used in (1) are unobserved in RNA microarray expression level measurements, proxies to them can be obtained using available microarray expression data by constructing interaction terms from observable targeting miRNA Ago RNA levels. This preserves a representation of the relevant miRNA biology leading to target cleavage while avoiding complications leading to model nonlinearities, such as seen in equilibrium points of typical chemical kinetics systems. We note that Ago RNA levels are proxies to (unobserved) protein levels. As discussed in Protocol S1, the microarray data was processed to approximate mRNA concentration levels. We assume that these levels are positively related to protein concentration, and so the interaction between Ago mRNA and targeting miRNA levels ought to be positively related to RISC concentration.

Model (2) refines the system biological elements in (1) and provides the beginnings of a formal statistical model. Let *i* index tissue sample, *j* index an m/miRNA pair, and consider that expression levels are measured on the logarithmic scale. Further, let *mRNA_i_^j^* represent the level of the putative target mRNA in the *i*th tissue sample of the *j*th pair; *Ago2_i_* and *Ago134_i_* be levels of Ago 2 and Ago 1, 3 and 4 (combined); *miRNA_i_^j^* and *miRNA_i_^−j^* be levels of the targeting miRNA in the *j*th pair and the combined levels from other miRNAs; and *ε_i_^j^* be a random error term assumed to be normally distributed. As suggested, proxies for the concentration of targeting RISCs composed of Ago 2 and Ago 1, 3 or 4 are obtained as products of *miRNA_i_^j^* and *Ago2_i_* or *Ago134_i_* respectively, and analogously for such RISCs composed of miRNAs not under explicit study.
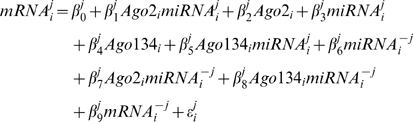
(2)


Under model (2), if the *j*th m/miRNA pair have a targeting relationship then β_1_ *^j^*<0 (indicating a negative relationship between expression levels of the mRNA and putatively targeting Ago 2 RISC proxy) would be anticipated. Therefore, a targeting relationship between the *j*th m/miRNA pair under consideration can be inferred by evaluating the no-targeting relationship hypothesis H_0_: β_1_ *^j^* = 0 vs. H_A_: β_1_ *^j^*<0. To contrast this approach with marginal expression level comparisons of mRNAs to miRNAs, note that an alternative to correlating m- and miRNA levels and evaluating the analogous no-targeting hypothesis H_0_: ρ*^j^* = 0 vs. H_A_: ρ*^j^*<0 (where ρ represents the true correlation level between m- and miRNA expression levels) would be to estimate the simple linear regression:
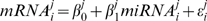
(3)and evaluate the hypothesis H_0_: β_1_ *^j^* = 0 vs. H_A_: β_1_ *^j^*<0. Of the other effect terms in (2), β_5_ *^j^* has arguably the most compelling physical interpretation - if the m/miRNA possess a targeting relationship (as evidenced by rejection of the no-targeting hypothesis), β_5_ *^j^* is anticipated to be positive and scaling in magnitude with β_1_ *^j^* due to the aforementioned competition for targeting sites between RISCs composed of Ago 2 and Ago 1, 3 or 4. The remainder of covariates and effects used in (2) are included to conform to statistical modeling standards that require inclusion of individual covariates in models that analyze interaction terms (e.g. *miRNA_i_^j^* and *Ago2_i_* terms), and to have a full representation of the variety of possible effects justified by the system biology (e.g. *Ago2_i_miRNA_i_^−j^*).

### Expression data

Regression models (2) and (3) were developed on and fit to data from two studies in which both human m- and miRNA expression levels were measured on a reasonably large set of tissue samples. A study of nasopharyngeal cancer (NPC) by researchers in Madison, WI and elsewhere [10],[42] derived whole genome Affymetrix hgu133plus2 microarrays for mRNA profiling, a custom cDNA array for miRNA profiling and RT-PCR for the expression of Epstein-Barr (EBV) genes. Data are available on 31 NPC and 10 normal tissue samples. The second data source was derived from that produced from a study of miRNA expression patterns over a wide variety of tumor and normal tissue types conducted by the Broad Institute [43]. This data collection measures m- and miRNA expression across 67 tissue samples from 10 different normal and tumor tissue types, each tissue type is represented by at least 5 sample observations. Additionally, we merged the Madison and Broad data to create a third dataset in order to fit (2) and (3) to data from the largest number of tissue samples possible. The merged dataset measured m- and miRNA expression across 108 tissue samples from 12 different normal and tissue types (the tissue states from the Madison dataset were not represented in the Broad study). Details of the Madison, Broad and combined data collections is provided in Protocol S1.

### Known target pairs

In order to validate the system biological regression model, the TarBase miRNA target database [44] was used to derive a set of m/miRNA target pairs that both had been previously validated through the use of gene mRNA and protein-specific techniques (such as PCR, luciferase reporters and immunoblotting) and were represented in the Madison and Broad datasets. (We did not include relationships that were supported by microarray data alone.) In total, there were 76 such m/miRNA target pairs that were commonly measured in both the Madison and Broad datasets and that fit the above criteria (these target pairs were used in the combined data analysis), and 23 additional pairs measured in the Madison data alone. See Table S1 for information pertaining to each of these m/miRNA target pairs.

We note that TarBase classifies target pairs into those reported to result in cleavage or translational repression. To assure that the known target pairs used in this study are competent for observable cleavage, we examined the original studies supporting their inclusion in TarBase. We found no reason to reject any of the pairs labeled in TarBase as resulting in mRNA cleavage as being so competent. However, simultaneous translational repression and cleavage of was demonstrated by a number of target pairs classified in TarBase as translationally repressive [25], and in other studies the use of only protein to miRNA comparisons could not justify such a distinction. Based on our examination of the supporting studies and underlying system biology (as previously described), we did not reject any of the known target pairs based on their TarBase cleavage/translational repression classification and instead regarded all target pairs as competent for Ago 2 RISC-mediated cleavage.

### Computationally predicted target pairs

To evaluate the performance of the system biological regression model on computationally predicted but unverified m/miRNA target pairs we used the results of sequence-based comparisons summarized in the miRBase [45]–[47] and TargetScan databases and expression data from the Madison dataset to derive a set of putative target pairs that met three criteria: 1) They were predicted by both miRBase and TargetScan simultaneously, rather than either database singularly; 2) The putative targeting miRNAs in the pairs under consideration were previously identified as differentially expressed between NPC and normal tissue samples [10]; 3) The putative targeted mRNAs in the pairs were those that had above median expression variability. These criteria were used to assure confidence in both the computational target predictions as well as the data used to verify them. The use of putative target pairs simultaneously predicted by both miRBase and TargetScan was motivated by the relatively low overlap between predicted target pairs from these databases – conditional on the miRNA under consideration, TargetScan averaged 301 predicted targets meeting criteria (2) and (3) and miRBase averaged 379, with 48 in common. Constraining the analysis to those pairs with differentially expressed miRNAs and targeted mRNAs with above average expression variability assured that there was sufficient variability in expression levels to permit a statistical analysis to be conducted. (Using mRNAs with above median mean expression rather than variability yielded no substantial differences in the results of our study.) In total, there were 874 putative m/miRNA target pairs that were evaluated using the Madison dataset. See Table S2 for the specific predicted target pairs studied.

### Model refinement

The Madison, Broad and combined data collections each exhibit a number of idiosyncratic data effects that might affect the ability to detect m/miRNA target pair relationships. Both Madison and Broad datasets consist of expression measurements from multiple tissue types with highly differentiated expression profiles not directly related to m/miRNA targeting. In the Madison data the tumor samples exhibit varying levels of EBV activity, which has been related to the up- and downregulation of a wide variety of genes both previously [48] and in the Madison data set [42]. In the Broad data, no measurements for Ago 3 expression are available. Finally, in addition to the idiosyncratic effects from the Madison and Broad datasets individually, the composition of the merged dataset from two data studies can be anticipated to introduce complications to even a marginal analysis of m/miRNA expressions.

To compensate for these issues, when analyzing target pair expressions isolated from the Madison data we added two covariates to model (2): a dichotomous variable representing tumor/normal tissue sample state and the expression of the EBV gene EBNA 1. When analyzing target pairs from the Broad data, we added a vector of dichotomous covariates representing tissue type to compensate for tissue type effects and substituted terms aggregating only Ago 1 and 4 for those using Ago 1, 3 and 4. In marginal analyses of the Madison and Broad datasets, no compensation for tissue state was made – as described below, introduction of similar dichotomous variables to model (3) had no effect on the substantive results of the marginal analyses. When analyzing target pairs from the combined dataset, we added to model (3) a dichotomous covariate that represented the dataset origin (Madison/Broad) of the observation under analysis, and added to model (2) the expression of the EBV gene EBNA 1, a vector of dichotomous covariates representing tissue state, and a dichotomous covariate that represented dataset origin.

### Model selection and hypothesis testing

As for (2) and (3), models that include idiosyncratic data covariates can be used to infer a targeting relationship for the *j*th m/miRNA pair by evaluating the suggested no targeting relationship hypothesis. Evaluation of such a hypothesis is typically performed via a t-test, and for marginal m/miRNA comparisons using any of the Madison, Broad or combined datasets this procedure is appropriate as the number of parameters are relatively low compared to the number of tissue samples available. However, the high parameterization of the system biological models motivated an alternative analysis based on AIC score minimization [49]. From a fully specified model containing both system biology and idiosyncratic data effects, minimum AIC submodels were computed and examined to determine whether the proxy variable to RISCs composed of Ago 2 and the putatively targeting miRNA was retained as a covariate, and if so, whether the effect of that variable was negative. For observational studies, estimated parameters in models selected for parsimony are sufficient to infer an effect of the associated covariate and so such cases were taken as rejections of the no targeting hypothesis. Additionally, we note that models selected by the AIC criterion can be regarded as implicitly passing a cross-validation test [50]. Therefore, there is a strong relationship between our technique and those that would be predicated upon dividing the data into training and validation sets (e.g. for developing a predictive model for mRNA expression, in which putatively targeting miRNAs might be evaluated as a potential predictor).

### Randomization controls

To evaluate the significance of the numbers of positive identifications we repeatedly applied the marginal and system biology-based regression model approaches to randomized control data, recording the number of m/miRNA pairs identified as targeting for each repetition. Two complementary randomization schemes were used. In the “no-targeting null” scheme miRNA expressions from each pair under study were permuted across tissue samples, holding the m/miRNA pairing constant – i.e. we condition on the set of m/miRNA expression levels in a given pair, but we randomize their association by resampling the observed miRNA levels. (This randomization was done separately in the two sources to preserve dataset-specific effects in the combined dataset analysis.) By contrast, in our “random pairs” scheme sets of non-targeting m/miRNA pairs were constructed by independently sampling unrelated m- and miRNAs from those under study (i.e. from the set of known target pairs), thus randomizing the pairings while holding the expression levels unchanged across tissue samples.

For the analysis of the known target pair data, multiple (1000) iterations of both randomization procedures were used to construct no-targeting null and random pairs distributions of numbers of positive identification. These distributions provided the basis for calculating p-values for the numbers of positively identified target pairs actually obtained by the marginal m/miRNA comparisons and system biology-based regression models. Under the no-targeting null randomization, dependency between m- and miRNA expressions is explicitly removed. Therefore the distributions of numbers of identifications across repetitions obtained from this procedure can be regarded to be what might be expected if none of the m/miRNA pairs under consideration had true targeting relationships, and the p-values correspond to tests of the hypothesis that the statistical procedure detects more targeting relationships that what would be anticipated if none of the m/miRNA pairs under consideration were bona fide target pairs. Further, the median numbers of identifications from the distributions can be used to infer measures of test specificity. We note that the random pairs procedure does not guarantee that the pairs under analysis do not have a targeting relationship (although known target pairs are rejected from those used in the method, it is possible that the m/miRNA pair is targeting but not yet verified as such), and so inflated numbers of identifications relative to what might be observed under the no-targeting null are expected. In other respects, the distribution and p-values of observed numbers of identifications against the random pairs distribution can be used in the same manner as those from the no-targeting null.

We performed two different analyses that verified the intuitions and results from our randomization tests on the known target pair data. To assure that our no-targeting null distributions were composed of a sufficient number of samples, we reconstructed no-targeting null distributions for the Madison data using 10000 iterations of the procedure described above (rather than the 1000 originally used), and recomputed p-values for the numbers of positive identifications obtained by the marginal and model-based procedures. These p-values were substantially identical to those obtained using 1000 iterations, and considering the heavy computational resources these procedures require we therefore constrained our analysis to the 1000 iteration case. Next, to verify our expectations regarding inflated numbers of identifications in the random pairs distribution we constructed a version of this distribution for the Madison data that was composed of randomly paired m/miRNA expressions taken from the full set of measurements (rather than the subset of m- and miRNAs involved in known target pairs), recomputed p-values as previously and compared these p-values to those originally obtained. In this analysis, no effort was made to remove known target pairs from those randomly sampled. The results of this version of the random pairs scheme are described below, however the test strongly verified our original intuitions.

In the analysis of the computationally predicted target pairs, we conditioned on miRNA and used no-targeting null distributions to obtain 95% upper bounds for the numbers of verifications that might observed from either the marginal comparisons or system biology-based models if none of the predicted m/miRNA target pairs were *bona fide*. The numbers obtained from the marginal and system biology-based methods on the actual data were then compared to these bounds to provide an indicator of the relative commonality of the results and an informal assessment of the specificity of the methods, analogous to those obtained on the known target pairs. The 95% upper bounds of the no-targeting null distributions were generated using 100 iterations – again, considering the computational resources required we regarded this number as sufficient to obtain a reasonable estimate of the 95% level.

### Implementation

We began by analyzing the expression data from known target pairs. Marginal m/miRNA comparisons were made initially in order to provide a performance baseline for the regression models that incorporated system biological covariates. For the Madison and Broad data analyses, we calculated Pearson correlation statistics on m/miRNA expression levels to measure negative marginal associations, and R^2^ statistics from the simple linear regression described in model (3) to determine the amount of variation in targeted mRNA expressions attributable to that of targeting miRNA. For the combined data analysis partial correlations of m/miRNA expression controlling for data source were calculated, and adjusted R^2^ statistics were computed to compensate for the increase in model complexity due to the introduction of the dichotomous data origin covariate. (A partial correlation is a measure of the amount of common variation between two variables after accounting for the effects of a set of related covariates on both. It is analogous to a standard marginal correlation between two variables, which does not account for covariate effects. An adjusted R^2^ statistic is a measure of model fit analogous to the standard R^2^ statistic that compensates for the number of covariates in the model. See [51], Chapter 7.10 and 7.7 for technical descriptions of the partial correlation and adjusted R^2^ statistic respectively.) In each data analysis using marginal methods, the total numbers of positive identifications of m/miRNA target pairing obtained from evaluation of the no-targeting hypothesis through a t-test at the 5% level were obtained. Next, the performance of the system biological regression model on the data from known target pairs was evaluated. Partial correlation statistics for pairs of targeted mRNAs and proxies to RISCs constructed of targeting miRNA and Ago 2, adjusted R^2^ statistics, and numbers of positive identifications of m/miRNA target pairing obtained from use of the minimum AIC submodel procedures on the versions of model (2) that included data idiosyncratic covariates were computed and compared to the analogous baselines from the marginal m/miRNA comparisons. The number of positive identifications were additionally evaluated using the randomization controls to assure that we obtained greater numbers of identifications than what would be expected under the null hypothesis of none of the m/miRNA pairs under analysis being a legitimate target pair.

We continued by analyzing the computationally predicted target pairs using the Madison dataset. For each putative target pair, the simple linear regression described in model (3) and the version of the system biology-based regression model (2) that incorporated idiosyncratic data effects was used to evaluate the no-targeting hypothesis through a t-test at the 5% level and the minimum AIC submodel procedure respectively. The results from the marginal procedure provided a baseline for evaluating the performance of the system biology-based regression model. The total numbers of verifications both the marginal and system biology-based procedures were conditioned on miRNA and compared directly to one another.

Our analyses were implemented as scripts in the R programming language [52], which were executed on Macintosh OS X computers with installations of R 2.8.0 (earlier versions of R were used at earlier stages in our analysis). Dataset S1 contains the scripts and associated data used to study the known target pairs in the Madison, Broad and combined datasets. Alternatively, the first author may be contacted to provide the archive directly. The archive is commented and can be used to provide further information regarding our procedures, or to rerun our analyses on any system with an R installation (available through the Comprehensive R Archive Network, http://cran.r-project.org). Please direct any questions regarding the archive to the first author.

## Results

### Marginal m/miRNA associations identify very few known target pairs

**Figure 2:**
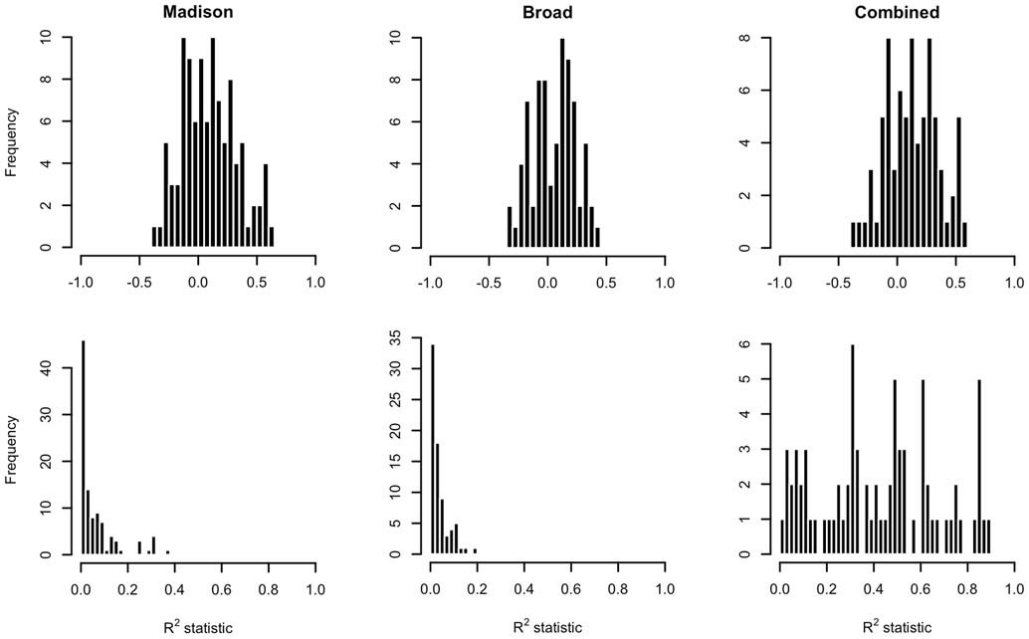
Marginal expression level comparisons of known target pairs. Marginal correlations of m- and miRNA expression levels are typically and inappropriately positive in all datasets under analysis. Further, in the Madison and Broad datasets the amount of variation in targeted mRNA expression captured by that of targeting miRNA is extremely low. In the combined dataset, high R^2^ values are indicative only of the amount of variation in mRNA expression due to data origin.

**Figure 3:**
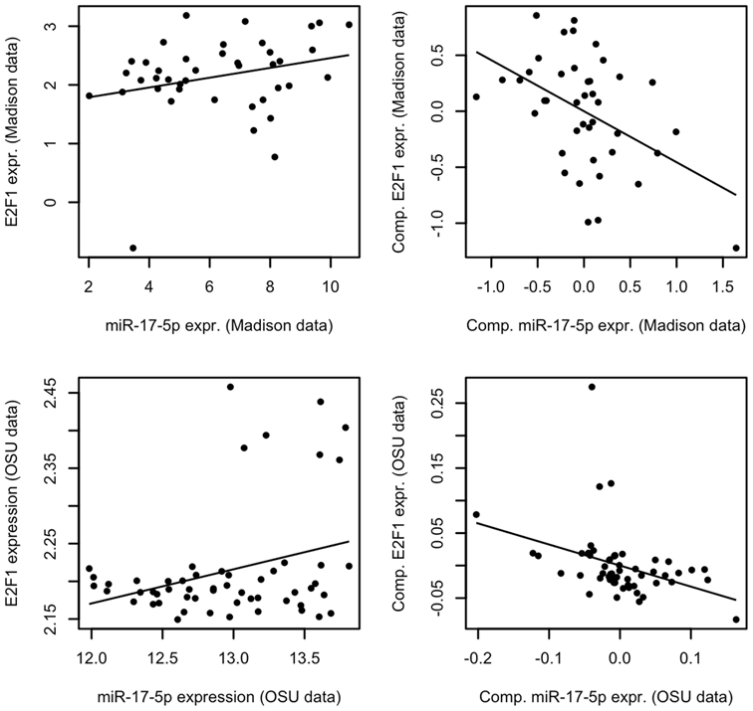
Marginal and system biology-compensated E2F1 and miR-17-5p expression levels. E2F1 and miR-17-5p expression levels in the Madison data are compared marginally in the top left and after compensation for biological and idiosyncratic covariates on the top right. Analogous results for the OSU data are provided in the bottom row. In either dataset, marginal RNA expression levels are not representative of the known targeting relationship between miR-17-5p and E2F1. After compensation the relationship between these RNAs can be observed.

Because the known target pairs under examination were previously observed to have targeting relationships, it was anticipated that the marginal correlations between m/miRNA expression levels using any of the Madison, Broad and combined datasets would typically be significantly negative. Contrary to these expectations, the sensitivity of marginal m/miRNA expression level comparisons was demonstrated to be quite low. Only 5 of the 99 target pairs in the Madison dataset, 6 of the 76 pairs in the Broad dataset and 7 of the 76 pairs in the combined dataset have significantly negative marginal relationships between m/miRNA expressions (Table 1), and the majority of observed correlations are positive (Figure 2). An example of the relationship between marginal m- and miRNA expression levels in the Madison data is provided in Figure 3 (top row, left column). The example provided compares miR-17-5p to E2F1, a known oncogene. Although miR-17-5p is known to target E2F1, the relationship between m- and miRNA levels is positive.

**Table 1:** Identification performances of marginal and regression models.

	Marginal comparison	AIC-optimal submodel
**Madison**	5/99 (5.05%)	33/99 (33.33%)
**Broad**	6/76 (7.89%)	20/76 (26.31%)
**Madison (Common subset)**	2/76 (2.63%)	27/76 (35.52%)
**Combined**	7/76 (9.21%)	36/76 (47.36%)

The AIC-optimal submodel procedures associated with regression models compensating for biological and idiosyncratic covariates capture more of the known targeting relationships than marginal m/miRNA expression level comparisons in all of the datasets under consideration. The Madison (Common subset) data refers to those target pairs in the Madison dataset which were in common with those in the Broad dataset.

As suggested in Methods, adding idiosyncratic data effects to our marginal m/miRNA comparison in (3) resulted in nearly no differences in the number of known m/miRNA target pairs successfully identified as such. Using t-testing procedures to evaluate the no-targeting hypothesis after doing so yields 3 of 99, 7 of 76 and 6 of 76 known m/miRNA target pairs identified as such in the Madison, Broad and combined datasets respectively. Similarly, a variety of data transformations were used to attempt to generate an improvement in the overall results without success, and the model fits were checked to assure that the results were not due to systemic outlier effects, model misspecifications or non-normal error terms.

Finally, it was notable that the number of detections obtained by marginal comparisons was well within what might be observed under either the no-targeting null or random pairs distributions (Figure 5, second row). For the analysis of the Madison data, the p-values of the number of positive identifications under the no-targeting null and random pairs distributions were 0.491 and 0.279 respectively, for the Broad data *p* = 0.193 and 0.800 respectively, and for the combined analysis *p* = 0.947 and 0.419 (Table 2). In the context of the previously discussed identification performance, these values suggest that the specificity of the marginal procedure approximates the false positive rate under the null hypothesis of no targeting, and therefore that marginal m/miRNA expression level comparisons are as likely to detect evidence of a targeting relationship for unrelated m- and miRNAs as they are for *bona fide* target pairs.

**Table 2:** Significances of numbers of correct identifications under randomization nulls.

	No-targeting null, marginal comparison	Random pairs, marginal comparison	No-targeting null, AIC-optimal submodel	Random pairs, AIC-optimal submodel
**Madison**	0.491	0.279	0.008	0.053
**Broad**	0.193	0.800	0.096	0.241
**Combined**	0.947	0.419	0.001	0.072

Table 2 provides p-values of the numbers of correct identifications for each of the Madison, Broad and combined datasets under no-targeting null and random pairs distributions. The number of correct identifications obtained after compensating for biological and idiosyncratic covariates is typically at least marginally significant in any of the datasets under study, using either randomization null. Marginal comparisons do not yield significant numbers of correct identifications in any of the cases under study.

### miRNA abundance alone explains only a small fraction of targeted mRNA variation

The observed R^2^ values from marginal m/miRNA expression level comparisons using data from known target pairs range from less than 0.001 to 0.365 with an average score of 0.061 for the pairs in the Madison data, less than 0.001 to 0.196 with an average of 0.035 for the pairs in the Broad data, and 0.008 to 0.880 with an average score of 0.411 for the pairs in the combined data (Figure 2). In the case of the Madison and Broad analyses, these values indicate that variation in the expression levels of targeting miRNAs explains only a small proportion of that in targeted mRNA levels. As a consequence of this, it would be anticipated that marginal comparisons of m/miRNA expression levels would not be useful for determining whether or not a targeting relationship exists, and therefore the low R^2^ values rationalize the previously observed performance of the marginal m/miRNA comparisons in identifying the known target pairs as such. In the case of the combined data analysis the observed R^2^ scores are substantially larger. However, the true relationships of the known m- and miRNA target pairs were not captured when analyzing the combined dataset with the marginal model. Therefore, these high R^2^ scores simply suggest that the majority of the observed variance in targeted mRNA expression is explained by the origin of the data observation, rather than the appropriateness of the model.

### Statistical models including system biological covariates better represent the known target pair data

**Figure 4:**
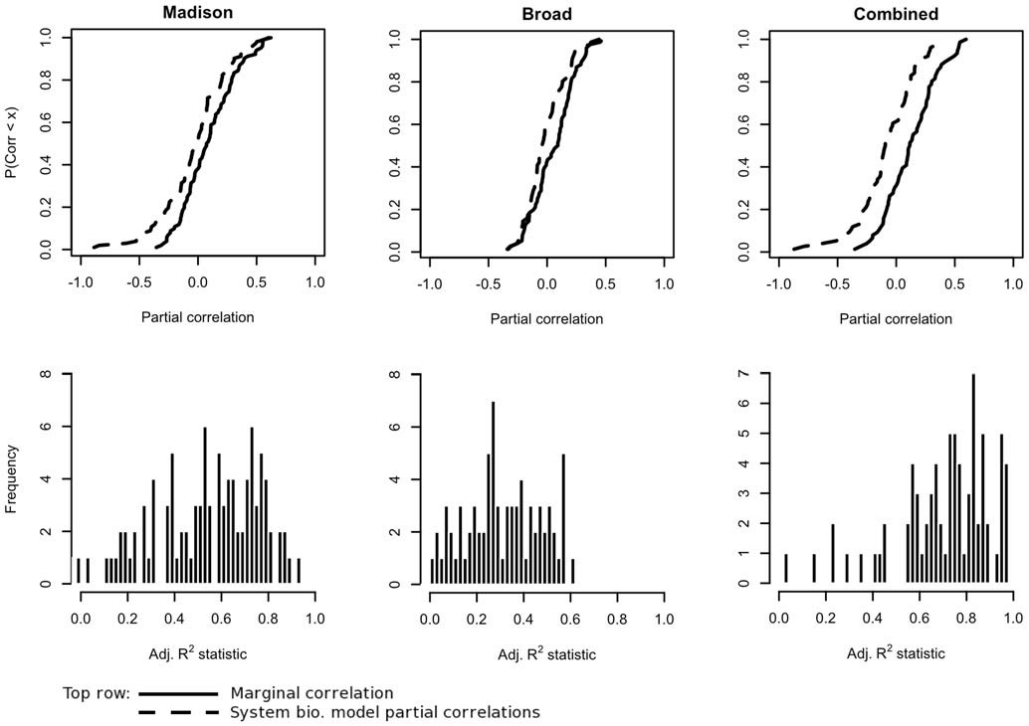
System biology-compensated expression level comparisons of known target pairs. Improvements in the percentages of variation in targeted mRNA expression are observed through use of the adjusted R^2^ statistic. Partial correlations of proxies to targeting Ago 2 RISCs and targeted mRNAs are lower than those of m/miRNA expressions. Paired Wilcoxon tests of the hypothesis H_0_: μ_0_−μ_1_ = 0 vs. H_A_: μ_0_−μ_1_≠0 were rejected for the Madison (*p* = 0.0135), Broad (*p* = 0.0186) and combined (*p*<0.0001) datasets, where μ_0_ and μ_1_ refer to mean correlations of marginal m/miRNA expression and mean partial correlations respectively.

The mean and range of adjusted R^2^ values for fits of the system biological regression model were (0.524, −0.102–0.922) for the Madison data, (0.310, −0.077–0.602) for the Broad data and (0.712, 0.055–0.974) for the combined data (Figure 4). The increases in observed R^2^ scores from the baselines obtained from fits of the marginal model indicate that the regression model captures a greater percentage of the variation in targeted mRNA levels, even after compensating for its increased complexity.

Because inclusion of the system biological covariates yielded a model that explained greater amounts of variation in target mRNA levels explained than the marginal model, it was anticipated that it would also better represent the true negative relationship between m/miRNA expression levels from the known target pairs. In fact, partial correlations between targeted mRNAs and proxies to targeting Ago 2 RISCs under model (2) were appropriately negative at substantially higher rates than marginal m/miRNA correlations (53% vs. 38%, 59% vs. 42% and 61% vs. 32% for the target pairs in the Madison, Broad and combined datasets respectively). Additionally, there was a reduction in observed correlation scores taken across the sample of m/miRNA target pairs (mean marginal and partial scores were (0.0934, −0.0247), (0.057, −0.012) and (0.232, −0.083) for the Madison, Broad and combined data). To formalize this comparison, a null hypothesis of equality of marginal and partial correlation scores was tested and rejected for all three datasets using a paired Wilcoxon rank-sum test (*p* = 0.014, 0.018 and <0.001 for the Madison, Broad and combined data).

Validation of these results consisted of checking the model fits for evidence of systemic outlier effects, model misspecifications or non-normal error terms, as was done for the marginal model fits. A comparative example of the model fits achieved in the Madison data is provided in the top row, right column of Figure 3. After controlling for system biological and idiosyncratic covariates, the relationship between miR-17-5p (which was positive under the marginal model) is appropriately negative.

In a further examination, the effects of the covariates used in the AIC-optimal submodels of the fits of (2) on the Madison data were studied to assure that the model was not overspecified. Of the variety of covariates used in the version of (2) compensating for the idiosyncratic data effects, only the dichotomous variable indicating tissue type found low levels of use in the AIC-optimal submodel – in fact, it was never included in the AIC-optimal submodels, indicating that tissue type never had a substantive effect on a targeted mRNA level after compensating for other effects. Because few if any of the known m/miRNA target pairs under consideration have been previously observed to be differentially expressed in NPC, this might be reasonable. Alternatively, this result can be explained by noting that EBV expression in the Madison data is highly associated with NPC, and therefore statistical control of EBV expressions rather than tissue type may be sufficient for both.

Related to this analysis, the estimated effects of proxies for targeting RISCs composed of Ago 1, 3 and 4 from the AIC-optimal submodels were compared to those composed of Ago 2 in order to assure that the model was performing in a reasonable manner. Figure 6 displays the relationships of estimated effects of targeting Ago 2 RISC proxies to targeting Ago 1, 3 and 4 RISC proxies, for AIC-optimal submodels estimated on the Madison data in which both covariates were included and the estimated effect of the targeting Ago 2 RISC covariate was appropriately negative (there were 13 such cases out of the 33 in which the effect of the targeting covariate was so). It can be observed that, as anticipated, the estimated effects of targeting Ago 1, 3 and 4 RISCs on targeted mRNA levels are indeed generally positive with effect sizes scaling with those of targeting Ago 2 RISCs.

Overall, these results demonstrate that the relationships between targeted mRNAs and proxies to targeting Ago 2 RISC, compensating for other relevant biological covariates, better represent the actual relationship of the known target pairs than marginal m/miRNA expression level correlations.

### m/miRNA associations compensating for the system biology identify a substantial portion of the known targets

Based on the improvements in model fit, it was further anticipated that evaluating the no-targeting hypothesis using the system biological model and the minimum AIC submodel procedure would indicate a greater number of positive identifications of targeting relationships than obtained by marginal m/miRNA comparisons. In fact, model (2) identified 33 of 99, 20 of 76 and 36 of 76 known m/miRNA target pairs as having expression profiles consistent with targeting relationships in the Madison, Broad and combined datasets respectively. This represents up to a sevenfold increase from the baseline obtained by marginal m/miRNA expression level comparisons (Table 1), and demonstrates the improved sensitivity of model (2) in detecting m/miRNA target pair relationships. We note that although under 50% of known target pairs were recovered by model (2), this level of identification performance is similar to the individual performances obtained by a number of sequence-based computational methods [30]. In particular, using the Madison and combined datasets we were able to successfully identify 33 and 47% of the known targets pairs we evaluated, whereas TargetScan and miRBase are reported to have 21 and 48% consistency with experimentally supported target pairs.

**Figure 5:**
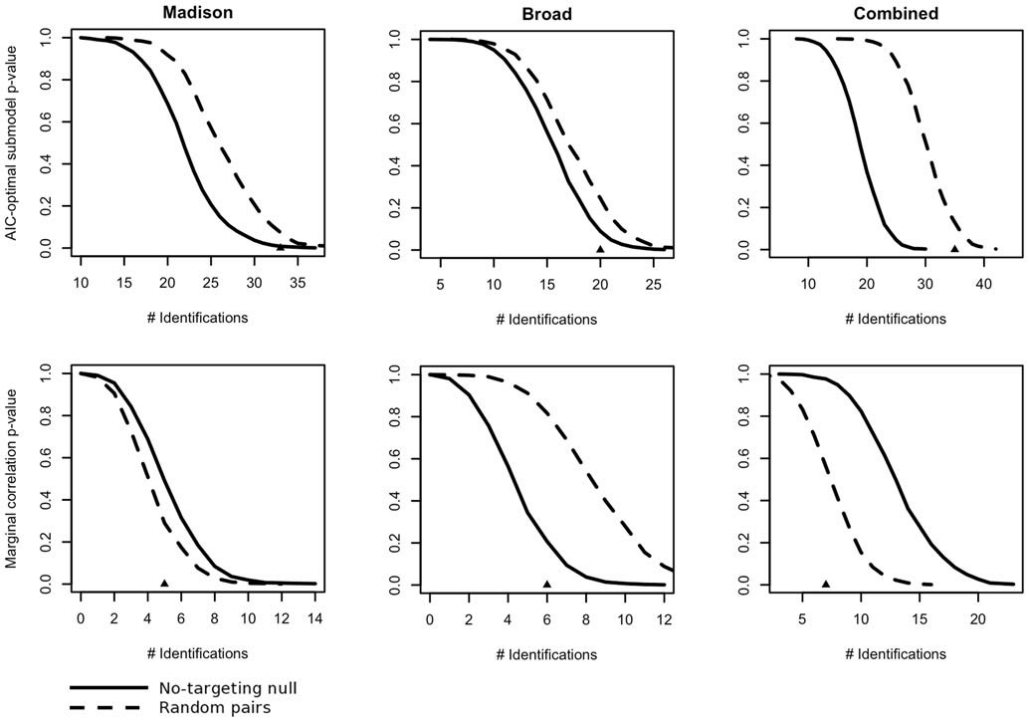
p-values of observed correct identifications under no-targeting and random pair nulls. The AIC-optimal procedure compensating for biological and idiosyncratic covariates yields numbers of correct identifications that are typically at least marginally significant in any of the datasets under study, using either randomization null. In no case is the number of correct identifications achieved by marginal correlation significantly greater than what would be expected.

The numbers of detections obtained by model (2) relative to what might be expected under either of the randomization techniques show similar improvements from the baseline obtained by marginal m/miRNA expression level comparisons (Figure 5, first row). For the analysis of the Madison, Broad and combined datasets, the p-values of the number of positive identifications under the no-targeting null were 0.008, 0.096 and 0.001 respectively. Likewise, under the random pairs distributions the p-values were 0.053, 0.241 and 0.072. In the case of the Madison, Broad and combined data analyses, the numbers of identifications obtained are at least marginally significantly greater than what is typically observed under the no-targeting null. Under the random pairs distribution the Madison and combined data analysis show similar results, while the number of identifications made using the Broad data is not significantly greater than what might be expected under the null. As suggested above we anticipated an overall inflation in p-values under the random pairs technique due to inadvertent sampling of as-of-yet unverified target pairs from the sets of known target pairs used as a basis for the technique. As discussed in *Methods*, to verify this intuition we performed a secondary analysis of the number of detections obtained for the Madison data against a random pairs distribution constructed from the full set of m- and miRNAs for which expression measurements were available. The p-value from this study was 0.007; based on this result we regarded the inflated p-values under the random pairs distributions as a statistical artifact and focused our attention on the results from the no-targeting null.

**Figure 6:**
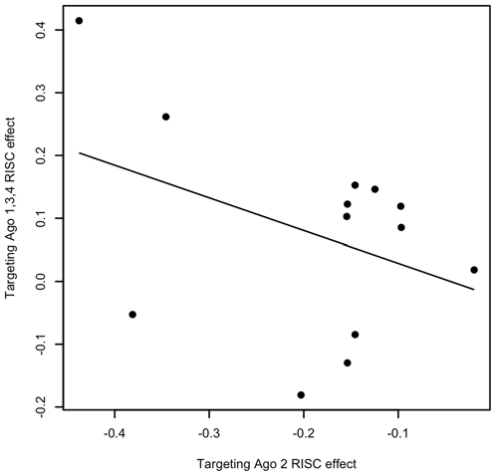
Estimated effects of targeting Ago 2 and Ago 1,3,4 RISC proxies. X- and y-axes correspond to estimated effects of targeting RISC proxies composed of Ago 2 (β_1_) and Ago 1, 3 or 4 (β_5_) on targeted mRNA levels for those cases where the model successfully identifies the known targeting pair and estimates both effects in the minimum AIC submodel. Observed values are inversely related in sign with similar magnitudes of effect strength. This is consistent with the hypothesized interference of observable target cleavage by RISCs composed of Ago 2 by those composed of Ago 1,3 or 4.

In total, the results from our analysis imply that the improvements in sensitivity for detecting target pairs obtained through model (2) are greater than any loss in specificity that might be incurred relative to that of the marginal procedure. The overall specificity of (2) for rejecting non-targeting pairs in the Madison and combined datasets are approximately 80%, as can be observed from median numbers of acceptances under the non-targeting null distribution. The analysis of the Broad data did not yield significantly larger numbers of correct identifications under either the no-targeting null or random pairs distributions, however it is useful to note that the high number of tissue types and missing Ago 3 measurements in the Broad dataset can be anticipated to negatively affect our ability to detect m/miRNA target pair relationships from expression levels. As well, the Madison dataset was processed to provide measurements in terms of concentration estimates that can more naturally be aggregated than the RMA measurements provided in the Broad data.

### Regression-based m/miRNA association verifies substantial portions of the predicted target pairs as *bona fide*

**Figure 7:**
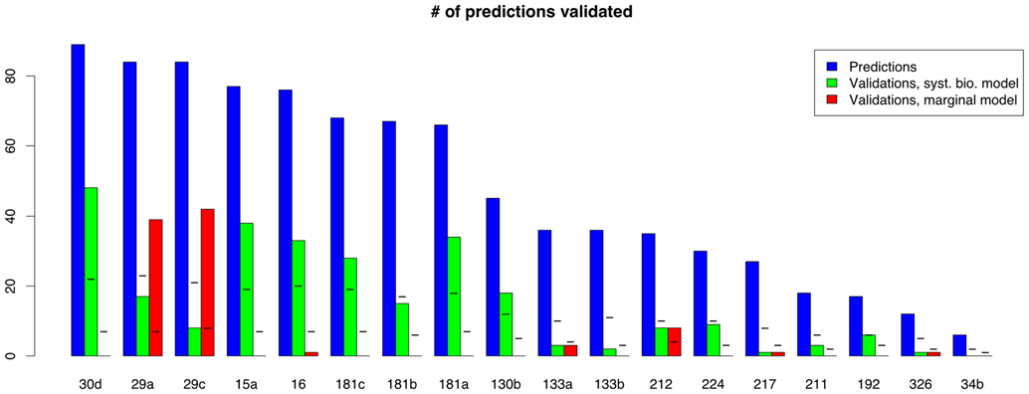
Validation of computationally predicted targets. Hash marks denote the 95% confidence level of identification numbers under the no-targeting null for the miRNA and validation technique under consideration. Overall, 7.83% of computational predicted targets were verified using marginal expression level comparisons, and 4 miRNAs showed substantially larger numbers of verifications than what would be typically expected. After compensating for biological and idiosyncratic effects, 25.68% of these targets were verified, and 9 miRNAs showed large numbers of identifications.

The overall results of evaluating the computationally predicted m/miRNA target pairs on the Madison data with the system biological regression model and marginal m/miRNA comparison are described in Figure 7. (Table S2 provides further detail on results obtained for particular m/miRNA pairs analyzed by the system biologic regression model.) For each miRNA under consideration, the first, second and third columns of Fig. 7 provide the numbers of putative target pairs evaluated and positive validations obtained by the system biological model and marginal m/miRNA comparisons respectively. In the second and third columns, 95% upper bounds on number of positive validations expected under the no-targeting null are provided.

Visual inspection of Figure 7 suggests that model (2) yields substantially more verifications than the marginal method in nearly every case. In fact, the marginal method most often yields no verifications of computationally predicted targets of any of the miRNAs considered. The average percentage of predicted targets validated by the system biologic regression model is 25.68%, taken across all miRNAs. For 6 of the 18 miRNAs conditioned upon, the number of verifications obtained was significantly (*p*<0.05) greater than what might be expected under the no-targeting null (miR-130b, -15a, -16, -181a, -181c, -30d), and analyses conducted on targets predicted for an additional three miRNAs (miR-192, -224 and -212) yielded numbers of identifications that were substantially greater (*p* = 0.08, 0.13 and 0.28 respectively). In comparison, marginal comparisons validate an average of 7.83% of predicted targets, and yielded three miRNAs (miR-212, -29a and –29c) associated with significantly greater numbers of verifications than what might be expected under the no-targeting null with one additional miRNA (miR-133a) having a substantially greater number (*p* = 0.21). Although some inflation in the number of verifications that might be observed under the no-targeting null was incurred through when using the system biologic regression model rather than marginal m/miRNA comparisons, the results obtained here are roughly consistent with the performance of the marginal and system biologic regression methods on the set of known target pairs.

**Figure 8:**
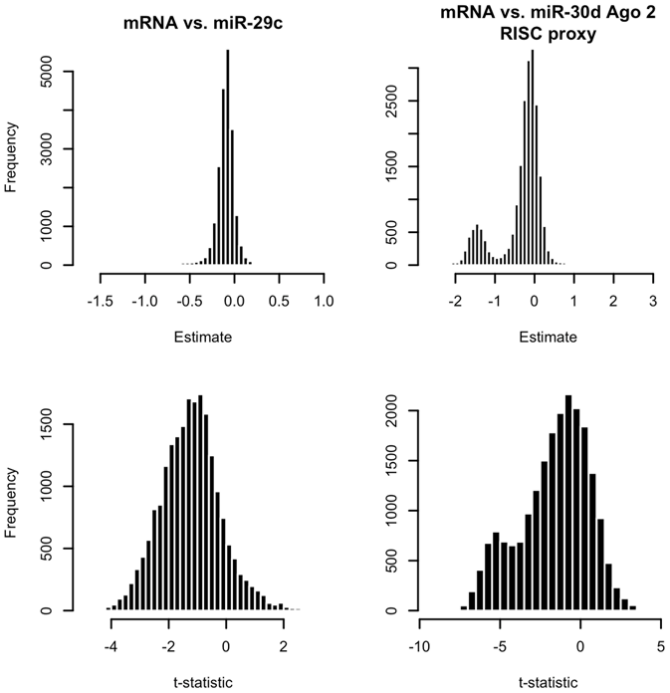
Estimated effects of marginal miR-29c expressions and miR-30d Ago 2 RISC proxies. Distributions of miR-30d Ago 2 RISC proxy effects across all genes measured in the Madison dataset suggest two classes of genes – those with no relationship to miR-30d, and those negatively regulated by miR-30d. Analogous distributions of marginal miR-29c expression effects suggest no such dichotomy, and are negatively biased.

Based on these results, a further comparative inspection was made of the distributions of the estimated marginal and Ago 2 mediated effects from fits of the miRNAs under analysis against all mRNAs in the Madison dataset. Sample distributions for estimated and normalized marginal effects of miR-29c and estimated miR-30d Ago 2 RISC effects are provided in Figure 8. (These were selected due to their high numbers of predicted target pair verifications, as seen in Figure 7.) The estimated marginal effects of miR-29c are clearly negatively biased, explaining the high numbers of validations. The estimated miR-30d Ago 2 RISC effects do not have such a bias. Instead, they demonstrate a bimodality with a main mass centered at 0 effect and a smaller mass centered at −1.5. Such a distribution is consistent with a categorization of genes into two classes: those regulated by miR-30d, and those not. Although analyses of such large-scale screen results are ongoing, the results in Figures 7 and 8 provide further evidence that that use of statistical models which compensate for the system biology related to miRNA-based gene silencing are more appropriate for validating and predicting m/miRNA targeting relationships than marginal expression level comparisons.

## Discussion

The effects of miRNAs on mRNA stability and translation are presently understood to have effects on organism development and physiological function, and have been linked to diseases such as cancer. It is of acknowledged importance to develop greater insight into the targeting relationships between m- and miRNAs. In this paper, we considered the role that biology-based statistical modeling and methods might play in the m/miRNA target prediction problem. Currently, the statistical techniques used for these purposes are typically based on marginal comparisons of individual m- and miRNA expressions across tissue samples. In some respects this is a natural comparison to consider – many early studies verifying predicted targeting relationships were based on transfection experiments with small numbers of samples, for which marginal m/miRNA comparisons might be the only procedure available. However, it has been observed previously (and was demonstrated here) that in practice these methods typically yield relatively disappointing results.

We hypothesized that improvements in the performance of statistical methods for detecting m/miRNA target pair relationships might be achieved through development of a statistical model and associated hypothesis testing procedure better tied to the underlying system biology. In an investigation of this biology in *homo sapiens* we identified a number of factors that we expected to affect the ability of marginal m/miRNA expression level comparisons to detect targeting relationships, many related to the dependence of the gene silencing mechanism on the construction and varied actions of RISCs. Based on this as well as additional information pertaining to the data under analysis, we developed regression methodology for testing hypotheses of no targeting relationship between m- and miRNA. Our rationale for choosing regression methods (as opposed to other possible statistical or computational methods) was motivated by the balance it offered between the competing goals of fidelity to the system biology, having a methodology with understood theoretical underpinnings and computational tractability for analyzing large number of putative m/miRNA target pairs, while being appropriate to the data quality and sample size.

In comparison to procedures based on marginal m/miRNA expressions, our models and procedures were shown to provide substantial improvements in overall model fit and detection performance for sets of known m/miRNA target pairs, although the degree of such improvement was somewhat dependent on the study design. As would be hoped, we further demonstrated that such improvements were carried over into the problem of validating predicted m/miRNA target pairs. Our study suggests that use of the regression models and associated hypothesis testing procedures developed here (or equivalent techniques based on the system biology) represent a reasonable alternative to methods based on marginal m/miRNA comparisons for analyzing expression data in m/miRNA targeting studies, and in conjunction with high throughput data can be used to either verify computationally predicted relationships or generate *de novo* information regarding m/miRNA target pairs. In fact, our model demonstrates consistency with known target pairs on par with many computational target prediction algorithms [30].

Because there have been few systematic studies of statistical methods for detecting m/miRNA targeting, there is little context that can be used to help evaluate our results. The most relevant external work is that recently conducted by Huang *et al* [53]–[55], however there are a number of differences between our studies. Huang *et al* focus on Bayesian methods to update a set of prior probabilities of targeting relationships between m- and miRNAs using marginal expression comparisons. These prior probabilities are, in their reported work, highly tied to the results of computational target prediction algorithms (in particular, TargetScan). The posterior probabilities obtained through their technique are compared to a threshold based on those obtained from a high-confidence set of m/miRNA target pair expression values; m/miRNA pairs with posterior targeting probabilities meeting the threshold are accepted as valid target pairs. In contrast, our study is framed in terms of evaluating a single m/miRNA pair for evidence of a targeting relationship, compensating for the underlying system biology (which includes the effects of other targeted and targeting m- and miRNAs on the m/miRNA pair under consideration). Our use of a hypothesis testing framework allows us to avoid the need to set a thresholding value based on a separate set of m/miRNA expression data for evaluating whether potential m/miRNA pairs evidence a targeting relationship. We do not tie our work to any particular computational target prediction algorithm, a position we view as appropriate given the issues with their specificity, sensitivity and inter-algorithm consistency.

Further, the emphasis of our presentation of algorithm development and results is substantially different from Huang *et al*. We choose to focus development of a statistical method on known m/miRNA pairs and then use the resulting procedure to validate a set of computational target predictions. Huang *et al* are primarily concerned with using their algorithm to validate computational predictions, with verification of their method on known target pairs taking place only on those that are represented in their set of computational target predictions [53]. It is unclear whether these differences in presentation have a substantial difference in performance. The methods proposed here and by Huang *et al* verify approximately the same proportion of computational target predictions evaluated, and Huang *et al* [53] demonstrate that of 19 known target pairs contained in the set of computationally predicted targets that they attempt to evaluate, 9 are identified as such. Overall, comparing the two methods and constructing new statistical procedures that incorporate elements of each may be one direction for achieving further improvements in the ability to detect m/miRNA target relationships from high-throughput expression data.

A similar issue that this study only indirectly addresses is the topic of how to best combine results across multiple sequence-based computational or expression-based methods, in order to obtain an aggregate estimate of the full set of m/miRNA target pairs occurring in humans. Such techniques can be classified into two categories: Those that would use sequence-based and expression-based methods sequentially (e.g. using expression-based methods to validate sequence-based predictions or using sequence-based methods to rationalize *de novo* expression-based predictions with a target site), and those that would use them simultaneously (i.e. without using one type of method conditional on the results of the other). Here, after establishing the utility of our data on known target pairs, we demonstrate how it might be used in a sequential study conditional on the results of sequence-based methods. To perform either a sequential study in which sequence-based methods are used conditional on *de novo* expression-based predictions or a simultaneous study using both sequence-based and expression-based predictions, the development of statistical methods which can distinguish between a bona fide m/miRNA target pair and m/miRNA pairs related through an intermediate, targeted, translationally activating mRNA must be developed. We are currently working on the development of such a technique. Additional complications that ought to be addressed in such studies is how best to handle the multiple comparisons problems that occur due to the large number of m/miRNA pairs that might be evaluated (which are orders of magnitude larger than those encountered in typical differential expression studies, for example), and how to best align results from multiple algorithms and datasets. We feel that, much as this study utilized known m/miRNA target pairs to validate our regression model, it is reasonable for future proposed methods for handling these technical problems to use them as a basis for evaluation and validation.

Aside from our current work towards the development of a statistical technique capable of *de novo* m/miRNA target pair prediction, we are extending our work in large-scale screening of putative m/miRNA target pairs (such as described in Figure 8). Our work consists of both investigating and improving our statistical procedures for inferring such relationships as well as aligning predictions from sequence- and expression-based methods, and by further supplementing the data used in this study with new samples as they become available. In a study of a recent dataset originally analyzed by Ambs *et al* [56], many of the results obtained here are reiterated. Figure 3 provides an example. Consistent with our result using the Madison data, miR-17-5p shows no substantial relationship with E2F1 in a marginal analysis (bottom left panel), but after controlling for the biological and idiosyncratic covariates the true negative relationship between them can be observed (bottom right). Based on this study, those of Huang *et al*, and the continued release of high-throughput data studies comparing m- and miRNA expression, we look forward to the further development of statistical methods for detecting m- and miRNA targeting relationships from expression data.

## Supporting Information

Protocol S1 Protocol
(0.05 MB DOC)

Table S1 Known m/miRNA target pairs. Table S1 contains the set of all previously observed target pairs used in this study. Alternative nomenclature for miRNAs/genes is provided. Targeted genes are labeled (C) or (TR) depending on whether the target pair's annotation in TarBase indicates previously observed evidence of mRNA cleavage or translational repression respectively. The citation provided by TarBase justifying the targeting relationship is also provided.
(0.06 MB DOC)

Table S2 Predicted m/miRNA target pairs. Table S2 contains the set of all predicted target pairs analyzed in this study. For each miRNA Table S2 provides the numbers of individual and simultaneous predictions made by miRBase and TargetScan for which the Madison dataset measured above noise expressions, along with a list of the genes analyzed. Predictions verified by use of model (2.3) are highlighted.
(0.04 MB DOC)

Dataset S1 Data and statistical codes. Expression data and supporting R codes for Stanhope et al (2009) “Statistical use of Argonaute expression and RISC assembly in microRNA target identification.”
(6.80 MB CDX)
