# Supplementary material for: Statistical Use of Argonaute Expression and RISC Assembly in microRNA Target Identification
Source: PLoS Comput Biol. 2009 Sep 25;5(9):e1000516. doi: 10.1371/journal.pcbi.1000516 (PMC2739424; doi:10.1371/journal.pcbi.1000516)
Supplement: Protocol S1 — Protocol (0.05 MB DOC) [file pcbi.1000516.s001.doc]

## Protocol S1

Two datasets were primarily used in the analyses conducted in this paper. The first was previously used in studies of nasopharyngeal cancer (NPC) at the University of Wisconsin – Madison [1,2]. The Madison data consists of 41 nasopharyngeal tissue samples, 31 of which are from NPC tumors and 10 from normal tissue. On each of these samples, three sets of measurements were taken. The first consists of an Affymetrix HG-U133-Plus 2 mRNA microarray, measuring the expressions of 54675 transcripts corresponding to approximately 38500 human genes. Next, the expressions of 207 different miRNA transcripts were measured using a custom-built miRNA microarray. Finally, PCR measurements of 10 different Epstein-Barr virus (EBV) genes were taken.

The raw data from the Affymetrix mRNA microarrays used in the Madison study were processed to obtain mRNA concentration estimates [3] in order to put the measurements in units natural for aggregation (such as done in our models). To obtain overall expression levels for a given gene in a tissue sample the concentration levels corresponding to each variant of mRNA produced by that gene were summed. Because the transformation from probe intensities to concentration estimates is based on spike in studies of an Affymetrix microarray (the Human HG-U95 Latin Square experiment conducted by Affymetrix) and no comparable work has been done on the miRNA microarray, the miRNA expression values previously determined by [2] were used directly in our comparisons of m/miRNA expressions. PCR expression measurements of the EBV gene EBNA 1 were normalized as in [1] and used directly in our system biologic models relating m/miRNA expression as a proxy for EBV activity. It was previously demonstrated that EBV is active in the NPC tissue samples used in this study and not in the normal, as measured by EBNA 1 expression [1].

The second dataset used in this study was previously used to analyze overall patterns of miRNA expression in a variety of cancers at the Broad Institute [4], and was obtained from the Broad website (http://www.broad.mit.edu/cgi-bin/cancer/datasets.cgi, “MicroRNA Expression Profiles Classify Human Cancers”). We analyzed 67 tissue samples from 10 different tissue types: 7 colon tumor, 8 pancreatic tumor, 6 bladder tumor, 6 normal prostate, 6 prostate tumor, 5 ovary tumor, 10 uterine tumor, 5 lung tumor, 8 mesothelioma and 6 breast tumor. (We note that the dataset contains additional tissue samples not used in this study because they were not sufficiently duplicated across tissue type to control for differences in mRNA expression attributable to tissue type differences rather than miRNA activity.) For each of the samples, mRNA expression measurements were made using the Affymetrix Hu6800 and Hu35KsubA microarrays (7129 and 8934 transcripts respectively) and the expressions of 151 human miRNAs were measured were made using a custom-built microarray. This data was not processed beyond aggregating the provided mRNA expressions into genes, as done for the Madison dataset.

The Madison and Broad datasets were combined to create a third dataset containing information on 10093 and 151 common m- and miRNAs taken over 108 tissue samples from 12 different tissue types. The expression of Ago 3 and the EBV gene EBNA 1 in observations originating in the Broad data were assigned the value 0. We note that no effort was made to process the Madison and Broad datasets with the same algorithm. Rather, we simply merged the dataset and compensated for the differences in our regression modeling.

The known miRNA-gene targeting relationships that are used in this study are reported in Table S1 and were taken from the TarBase miRNA target database [5]. There are 76 target pairs commonly observed in both the Madison and Broad datasets, and 23 additional pairs observed in the Madison data alone. The description of each pair in Table S1 consists of the names of the miRNA and the targeted gene (and any alternative nomenclature); the targeting relationship category reported by TarBase (cleaving / translationally repressive); a citation to the originating study; and those computational target prediction databases (miRBase [6-8]; PicTar [9]; TargetScan [10-12]) that contain a prediction consistent with the known relationship. TarBase suggests more previously observed targeting relationships than reported in Table S1. Targeting relationships suggested by TarBase that involved an m- or miRNA for which no data were collected or were suggested by solely microarray studies were discarded. The targeting relationships described are those that were determined through the use of gene mRNA and protein-specific techniques such as PCR, luciferase reporters and immunoblotting. TarBase distinguishes between cleaving and translationally repressive targeting relationships, and we report this information to be consistent with its contents. However, we examined the original studies supporting the 16 target pairs identified as cleaving that were included in our analysis to verify this categorization. To provide some context to this examination, we also analyzed studies supporting the 83 targeting relationships cited in TarBase as resulting in translational repression of the targeted mRNA. We did not find any reason to reject any of the target pairs labeled in TarBase as resulting in mRNA cleavage. However, simultaneous translational repression and cleavage of miRNA targets was demonstrated by a number of the studies supporting target pairs cited in TarBase as translationally repressive [13], and in other studies the use of only protein to miRNA comparisons could not justify such a distinction. Therefore, we regard this category as noninformative.

In extracting data for the previously observed target pairs from either the Madison, Broad or combined datasets, the gene and miRNA targeting relationships reported in TarBase did not always refer to genes and miRNAs in a consistent manner with their labels on the m- and miRNA microarrays. The gene and miRNA titles given in Table S1 are from TarBase. If there was a mismatch between these titles and those used by the microarray devices, the title used on the device is appended to the title in parentheses. In some cases, a unique relationship between the miRNA title used by TarBase and the miRNA microarray could not be determined. In such cases, the set of miRNA microarray probes corresponding to the miRNA indicated by TarBase are reported, and the miRNA expression was obtained by summing the individual probe expressions.

To verify that the known target pairs were substantially expressed in the tissue samples from the Madison dataset, we checked the mean, variance and range of RMA expression values for each probe corresponding to each gene and miRNA under analysis across the tissue samples in each data set. The effects of gene/miRNA nonexpression on the results described in this paper are minimal. Each of the 99 genes under analysis were associated with at least one probe with a RMA expression level greater than 5.0, and only 6 of the pairs were associated with a gene for which all probes were less than 6.0 (RMA values of approximately 5.0-6.0 are typically regarded as measurable response). Similar checks and aggregation were done for Argonaute gene expression levels. We found the observed expression levels for Ago 1-4 to be above noise levels and varying substantially. Although there is no minimal threshold for the expression levels from the miRNA microarray used in this study analogous to that described for mRNA microarrays processed by RMA, a simple check was done by generating mean expression values for each of the 220 miRNAs under study and comparing the means of those used in the target pairs under study to those of the entire population. All of the miRNAs under analysis were ranked in the top 90% of mean expressions. No substantial effort was made to verify high expression of the known target pairs in the Broad data, however the observed expression levels for Ago 1-4 were previously found to be above noise levels and varying substantially across tissue samples [4].

The computationally predicted miRNA-gene targeting relationships that are used in this study are reported in Table S2. There a total of 873 pairs predicted simultaneously in the miRBase and TargetScan databases corresponding to measurements in the Madison data with differentially expressed miRNAs and mRNAs with above average expression variability. (These criteria were applied to assure high-confidence computational predictions and above noise measurements.) For each miRNA, Table S2 provides the numbers of individual and simultaneous predictions from miRBase and TargetScan and a list of the simultaneously predicted gene targets, with those verified highlighted in red.

## References

1. Sengupta S, den Boon J, Chen I-H, Newton M, Dahl D *et al*. (2006) Genome-Wide Expression Profiling Reveals EBV-Associated Inhibition of MHC Class I Expression in Nasopharyngeal Carcinoma. Cancer Res 66 (16): 7999-8006.
2. Sengupta S, den Boon J, Chen I-H, Newton M, Stanhope S *et al*. (2008) MicroRNA 29c is Downregulated in Nasopharyngeal Carcinomas, Upregulating mRNAs Encoding Extracellular Matrix Proteins. Proc Natl Acad Sci U S A 105 (15): 5874-5878
3. Hekstra D, Taussig A, Magnasco M, Naef F (2003) Absolute mRNA concentrations from sequence-specific calibration of oligonucleotide arrays. Nucleic Acids Res 31 (7): 1962-1968.
4. Lu J, Getz G, Miska E, Alvarez-Saavedra E, Lamb J *et al*. (2005) MicroRNA expression profiles classify human cancers. Nature 435 (9): 834-838.
5. Seupathy P, Corda B, Hatziegeorgiou A (2006) TarBase: A comprehensive database of experimentally supported animal microRNA targets. RNA 12: 192-197.
6. Griffiths-Jones S (2004) The microRNA Registry. Nucleic Acids Res 32: D109-D111.
7. Griffiths-Jones S, Grocock R, van Dongen S, Bateman A, Enright A (2006) miRBase: microRNA sequences, targets and gene nomenclature. Nucleic Acids Res 34: D140-D144.
8. Griffiths-Jones S, Saini H, van Dongen S, Enright A (2008) miRBase: tools for microRNA genomics. Nucleic Acids Res 36: D154-D158.
9. Krek A, Grun D, Poy M, Wolf R, Rosenberg L *et al*. (2005) Combinatorial microRNA target predictions. Nat Genet 37 (5): 495-500.
10. Lewis B, Shih I, Jones-Rhoades M, Bartel D, Burge C (2003) Prediction of Mammalian MicroRNA Targets. Cell 115: 787-798.
11. Lewis B, Burge C, Bartel D (2005) Conserved Seed Pairing, Often Flanked by Adenosines, Indicates that Thousands of Human Genes are MicroRNA Targets. Cell 120: 15-20.
12. Grimson A, Farh K, Johnson W, Garrett-Engele P, Lim L *et al*. (2007) MicroRNA Target Specificity in Mammals: Determinants beyond Seed Pairing. Mol Cell 27 (1): 91-105.
13. Nakamoto M, Jin P, O’Donnell W, Warren S (2005) Physiological identification of human transcripts translationally regulated by a specific microRNA. Hum Mol Genet 14 (24): 3813-3821.
14. Kiriakidou M, Nelson P, Kouranov A, Fitziev P, Bouyioukos C *et al*. (2004) A combined computational-experimental approach predicts human microRNA targets. Genes Dev 18: 1165-1178.
15. Kawasaki H, Taira K (2005) Identification of target genes of microRNAs in retinoic acid-induced neuronal differentiation. Pure Appl Chem 77 (1): 313-318.
16. O’Donnell K, Wentzel E, Zeller K, Dang C, Mendell J (2005) c-Myc-regulated microRNAs modulate E2F1 expression. Nature 435: 839-843.
17. Esau C, Kang X, Peralta E, Hanson E, Marcusson E *et al*. (2004) MicroRNA-143 Regulates Adipocyte Differentiation. J Biol Chem 279 (50): 52361-52365.
18. Zhao Y, Samal E, Srivastava D (2005) Serum response factor regulates a muscle-specific microRNA that targets Hand2 during cardiogenesis. Nature 436 (14): 214-220.
19. Johnson S, Grosshans H, Shingara J, Byrom M, Jarvis R *et al*. (2005) RAS Is Regulated by the let-7 MicroRNA Family. Cell 120: 635-647.
20. Cimmino A, Calin G, Fabbri M, Iorio M, Ferracin M *et al*. (2005) miR-15 and miR-16 induce apoptosis by targeting BCL2. Proc Natl Acad Sci U S A 102 (39): 13944-13949.
21. Vo N, Klein M, Varlamova O, Keller D, Yamamoto T *et al*. (2005) A cAMP-response element binding protein-induced microRNA regulates neuronal morphogenesis. Proc Natl Acad Sci U S A 102 (45): 16426-16431.
22. Fazi F, Rosa A, Fatica A, Gelmetti V, De Marchis M *et al*. (2005) A Minicircuitry Comprised of MicroRNA-223 and Transcription Factors NFI-A and C/EBPα Regulates Human Granulopoiesis. Cell 123: 819-831.
23. Felli N, Fontana L, Pelosi E, Botta R, Bonci D *et al*. (2005) MicroRNAs 221 and 222 inhibit normal erythropoiesis and erythroleukemic cell growth via kit receptor down-modulation. Proc Natl Acad Sci U S A 102 (50): 18081-18086.
24. Chen J, Mandel E, Thomson J, Wu Q, Callis T *et al*. (2006) The role of microRNA-1 and microRNA-133 in skeletal muscle proliferation and differentiation. Nat Genet 38 (2): 228-233.
25. Volinia S, Calin G, Liu C, Ambs S, Cimmino A *et al*. (2006) A microRNA expression signature of human solid tumors defines cancer gene targets. Proc Natl Acad Sci U S A 103 (7): 2257-2261.
26. Garzon R, Pichiorri F, Palumbo T, Iuliano R, Cimmino A *et al*. (2006) MicroRNA fingerprints during human megakaryocytopoiesis. Proc Natl Acad Sci U S A 103 (13): 5078-5083.
27. Anderson P, Kedersha N (2006) RNA Granules. J Cell Biol 172 (6): 803-808.
28. Pekarsky Y, Santanam U, Cimmino A, Palamarchuk A, Efanov A *et al*. (2006) Tcl1 Expression in Chronic Lymphocytic Leukemia Is Regulated by miR-29 and miR-181. Cancer Res 66 (24): 11590-11593.
29. Chang J, Nicolas E, Marks D, Sander C, Lerro A *et al*. (2004) miR-122, a Mammalian Liver-Specific microRNA, is Processed from hcr mRNA and May Downregulate the High Affinity Cationic Amino Acid Transporter CAT-1. RNA Biol 1 (2): 106-113.
30. Fukuda Y, Kawasaki H, Taira K (2005) Exploration of human miRNA target genes in neuronal differentiation. Nucleic Acids Symp Ser 49: 341-342.
31. Scott G, Goga A, Bhaumik D, Berger C, Sullivan C *et al*. (2007) Coordinate Supression of ERBB2 and ERBB3 by Enforced Expression of Micro-RNA miR-125a. J Biol Chem 282 (2): 1479-1486.
32. Boutz P, Chawla G, Stiolov P, Black D (2007) MicroRNAs regulate the expression of the alternative splicing factor nPTB during muscle development. Genes Dev 21 (1): 71-84.
33. Martin M, Lee E, Buckenberger J, Schmittgen T, Elton T (2006) MicroRNA-155 Regulates Human Angiotensin II Type 1 Receptor Expression in Fibroblasts. J Biol Chem 281 (27): 18277-18284.
34. Meng F, Henson R, Lang M, Wehbe H, Maheshwari S *et al*. (2006) Involvement of Human Micro-RNA in Growth and Response to Chemotherapy in Human Cholangiocarcinoma Cell Lines. Gastroenterology 130: 2113-2129.
35. Saito Y, Liang G, Egger G, Friedman J, Chuang J *et al*. (2006) Specific activation of microRNA-127 with downregulation of the proto-oncogene BCL6 by chromatin-modifying drugs in human cancer cells. Cancer Cell 9 (6): 435-443.
36. Tuddenham L, Wheeler G, Ntounia-Fousara S, Waters J, Hajihosseini M *et al*. (2006) The cartilage specific microRNA-140 targets histone deacetylase 4 in mouse cells. FEBS Lett 580: 4214-4217.
37. Hossain A, Kuo M, Saunders G (2006) Mir-17-5p Regulates Breast Cancer Proliferation by Inhibiting Translation of AIB1 mRNA. Mol Cell Biol 26 (21): 8191-8201.
38. Tsuchiya Y, Nakajima M, Takagi S, Taniya T, Yokoi T (2006) MicroRNA Regulates the Expression of Human Cytochrome P450 1B1. Cancer Res 66 (18): 9090-9098.
39. Rosenberg M, Georges S, Asawachaicharn A, Analau E, Tapscott S (2006) MyoD inhibits Fstl1 and Utrn expression by inducing transcription of miR-206. J Cell Biol 175 (1): 77-85.
40. Wang X, Wang X (2006) Systematic identification of microRNA functions by combining target prediction and expression profiling. Nucleic Acids Res 34 (5): 1646-1652.
41. Hebert C, Norris K, Scheper M, Nikitakis N, Sauk J (2007) High mobility group A2 is a target for miRNA-98 in head and neck squamous cell carcinoma. Mol Cancer 6: 5.
42. Poy M, Eliasson L, Krutzfeldt J, Kuwajima S, Ma X *et al*. (2004) A pancreatic islet-specific microRNA regulates insulin secretion. Nature 432: 226-230.
43. Yekta S, Shih I, Bartel D (2004) MicroRNA-Directed Cleavage of HOXB8 mRNA. Science 304: 594-596.
44. Voorhoeve P, le Sage C, Schrier M, Gillis A, Stoop H *et al*. (2006) A Genetic Screen Implicates miRNA-372 and miRNA-373 As Oncogenes in Testicular Germ Cell Tumors. Cell 124: 1169-1181.
45. Abelson J, Kwan K, O’Roak B, Baek D, Stillman A *et al*. (2005) Sequence Variants in SLITRK1 Are Associated with Tourette’s Syndrome. Science 310: 317-320.
46. Wu L, Belasco J (2005) Micro-RNA Regulation of the Mammalian lin-28 Gene during Neuronal Differentiation of Embryonal Carcinoma Cells. Mol Cell Biol 25 (1): 9198-9208.
