# Supplementary material for: Statistical Use of Argonaute Expression and RISC Assembly in microRNA Target Identification
Source: PLoS Comput Biol. 2009 Sep 25;5(9):e1000516. doi: 10.1371/journal.pcbi.1000516 (PMC2739424; doi:10.1371/journal.pcbi.1000516)
Supplement: Table S1 — Known m/miRNA target pairs. Table S1 contains the set of all previously observed target pairs used in this study. Alternative nomenclature for miRNAs/genes is provided. Targeted genes are labeled (C) or (TR) depending on whether the target pair's annotation in TarBase indicates previously observed evidence of mRNA cleavage or translational repression respectively. The citation provided by TarBase justifying the targeting relationship is also provided. (0.06 MB DOC) [file pcbi.1000516.s002.doc]

**Table S1: Known m/miRNA target pairs.**

**Available in both Madison and Broad datasets**

| **miRNA (Alt. Nomenclature)** | **Targeted Gene (TarBase Ann.)** | Citation | Computational Predictions |
| --- | --- | --- | --- |
| miR-124 (miR-124a) | Mtpn (TR) | [9] | PicTar,TargetScan |
| let-7b | Mtpn (TR) | [9] | PicTar,TargetScan |
| miR-141 | Clock (TR) | [14] | miRBase |
| miR-24 | MAPK14 (TR) | [14] |  |
| miR-145 | FLJ13158 (PARP8) (TR) | [14] | MiRBase |
| miR-23a | FLJ13158 (PARP8) (TR) | [14] |  |
| let-7e | SMC1L1 (TR) | [14] |  |
| miR-15 (miR-15a) | DMTF1 (TR) | [14] | miRBase,PicTar,TargetScan |
| miR-199b | LAMC2 (TR) | [14] |  |
| miR-103-1 (miR-103) | FBXW1B (FBXW11) (TR) | [14] |  |
| miR-23 (miR-23a) | HES1 (TR) | [15] | PicTar |
| miR-20 | E2F1 (TR) | [16] | PicTar,TargetScan |
| miR-17-5p | E2F1 (TR) | [16] | MiRBase,PicTar,TargetScan |
| miR-30a-3p | vezatin (TR) | [13] | PicTar |
| miR-143 | ERK5 (MAPK7) (TR) | [17] | MiRBase |
| miR-1 | Hand2 (TR) | [18] | PicTar,TargetScan |
| miR-1 | TMSB4X (TR) | [18] |  |
| miR-23 (miR-23a) | POU4F2 (TR) | [10] | MiRBase,PicTar,TargetScan |
| miR-101 | Enx-1 (EXH1,2) (TR) | [10] |  |
| miR-101 | MYCN (TR) | [10] | MiRBase,PicTar,TargetScan |
| miR-19a | PTEN (TR) | [10] | TargetScan |
| miR-34 (miR-34c) | Notch1 (TR) | [10] | MiRBase,PicTar,TargetScan |
| miR-1b (miR-1) | G6PD (TR) | [10] | MiRBase,TargetScan |
| miR-1b (miR-1) | BDNF (TR) | [10] | TargetScan |
| miR-130 (miR-130a, -130b) | MCSF (CSF1) (TR) | [10] | PicTar,TargetScan |
| miR-26 (miR-26a, -26b) | SMAD1 (TR) | [10] | MiRBase,TargetScan |
| let-7 (-7a, -7b, -7c, -7d,-7e, -7f) | KRAS (TR) | [19] |  |
| let-7 (-7a, -7b, -7c, -7d,-7e, -7f) | NRAS (TR) | [19] | MiRBase,TargetScan |
| miR-124 (miR-124a) | MAPK14 (TR) | [9] | PicTar,TargetScan |
| miR-15 (miR-15a) | BCL2 (TR) | [20] | PicTar,TargetScan |
| miR-16 | BCL2 (TR) | [20] | PicTar,TargetScan |
| miR-30a-3p | tmem2 (TR) | [13] |  |
| miR-30a-3p | thbs1 (TR) | [13] |  |
| miR-30a-3p | slc7a6 (TR) | [13] |  |
| miR-30a-3p | pro2730 (TMEM113) (TR) | [13] |  |
| miR-30a-3p | tuba3 (TR) | [13] | MiRBase |
| miR-30a-3p | cyr61 (TR) | [13] |  |
| miR-30a-3p | cdk6 (TR) | [13] |  |
| miR-132 | RICS/p250GAP (TR) | [21] | PicTar |
| miR-223 | NFIA (TR) | [22] | MiRBase,PicTar,TargetScan |
| miR-221 | KIT (TR) | [23] | TargetScan |
| miR-222 | KIT (TR) | [23] | TargetScan |
| miR-133 (miR-133a) | SRF (TR) | [24] |  |
| miR-26a | PLAG1 (TR) | [25] | PicTar,TargetScan |
| miR-20a (miR-20) | TGFBR2 (TR) | [25] | TargetScan |
| miR-106a | RB1 (TR) | [25] | PicTar,TargetScan |
| miR-130 (miR-130a, -130b) | MAFB (TR) | [26] | PicTar,TargetScan |
| miR-10a | HOXA1 (TR) | [26] | PicTar,TargetScan |
| miR-1 | GJA1 (TR) | [27] | PicTar,TargetScan |
| miR-29 (miR-29a) | Tcl1 (Tcl1a) (TR) | [28] | MiRBase |
| miR-181 (miR-181a, -181b) | Tcl1 (Tcl1a) (TR) | [28] | MiRBase |
| miR-122 (miR-122a) | CAT-1 (SLC7A1) (TR) | [29] | PicTar,TargetScan |
| miR-23b | Notch1 (TR) | [30] |  |
| miR-24 | Notch1 (TR) | [30] |  |
| miR-27b | Notch1 (TR) | [30] |  |
| miR-125a | ERBB2 (TR) | [31] | MiRBase |
| miR-125a | ERBB3 (TR) | [31] |  |
| miR-133 (miR-133a) | PTBP2 (TR) | [32] | PicTar,TargetScan |
| miR-155 | AGTR1 (TR) | [33] | MiRBase |
| miR-21 | PTEN (TR) | [34] |  |
| miR-127 | BCL6 (TR) | [35] | miRBase |
| miR-140 | HDAC4 (TR) | [36] | PicTar,TargetScan |
| miR-17-5p | AIB1 (NCOA3) (TR) | [37] | PicTar,TargetScan |
| miR-27b | CYP1B1 (TR) | [38] | PicTar,TargetScan |
| miR-206 | Fstl1 (TR) | [39] |  |
| miR-206 | Utrn (TR) | [39] | MiRBase,TargetScan |
| miR-206 | GJA1 (TR) | [27] | PicTar,TargetScan |
| miR-124 (miR-124a) | VAMP3 (C) | [40] | PicTar,TargetScan |
| miR-124 (miR-124a) | ACAA2 (C) | [40] |  |
| miR-124 (miR-124a) | SLC16A1 (C) | [40] | PicTar,TargetScan |
| miR-124 (miR-124a) | LAMC1 (C) | [40] | PicTar,TargetScan |
| miR-124 (miR-124a) | CD164 (C) | [40] | PicTar,TargetScan |
| miR-124 (miR-124a) | SURF4 (C) | [40] | PicTar,TargetScan |
| miR-124 (miR-124a) | GAS2L1 (C) | [40] | PicTar,TargetScan |
| miR-124 (miR-124a) | ELOVL5 (C) | [40] | PicTar,TargetScan |
| miR-124 (miR-124a) | SUCLG2 (C) | [40] | PicTar,TargetScan |
| miR-124 (miR-124a) | NM_014445 (SERP1) (C) | [40] | PicTar,TargetScan |
| miR-98 (miR-124a) | HMGA2 (C) | [41] | PicTar,TargetScan |

#### Available in Madison dataset only

| **miRNA (Alt. Nomenclature)** | **Targeted Gene (TarBase Ann.)** | Citation | Computational Predictions |
| --- | --- | --- | --- |
| miR-375 | Mtpn (TR) | [42] | PicTar,TargetScan |
| let-7b | Lin28(TR) | [14] | PicTar,TargetScan |
| miR-16 | CGI-38 (TR) | [14] | MiRBase,TargetScan |
| miR-196 (miR-196a) | HOXA7 (TR) | [43] | TargetScan |
| miR-196 (miR-196a) | HOXC8 (TR) | [43] | MiRBase,PicTar,TargetScan |
| miR-196 (miR-196a) | HOXD8 (TR) | [43] | MiRBase |
| miR-34 (miR-34c) | DLL1 (TR) | [10] | MiRBase,PicTar,TargetScan |
| miR-23a | CXCL12 (TR) | [10] | PicTar,TargetScan |
| miR-375 | Jak2 (TR) | [9] | TargetScan |
| miR-375 | C1qbp (TR) | [9] | MiRBase |
| miR-375 | Usp1 (TR) | [9] | PicTar,TargetScan |
| miR-375 | Adipor2 (TR) | [9] | PicTar,TargetScan |
| miR-1 | HDAC4 (TR) | [24] | PicTar,TargetScan |
| miR-372 | LATS2 (TR) | [44] | PicTar,TargetScan |
| miR-373 | LATS2 (TR) | [44] | PicTar,TargetScan |
| miR-189 | SLITRK1 (TR) | [45] | MiRBase,PicTar |
| miR-124 (miR-124a) | ATP6V0E (C) | [40] | TargetScan |
| miR-196 (miR-196a) | HOXB8 (C) | [43] | MiRBase |
| miR-125a | Lin28 (C) | [46] | TargetScan |
| miR-125b | Lin28 (C) | [46] | TargetScan |
| miR-124 (miR-124a) | NM_018211 (RAVER2) (C) | [40] | TargetScan |

m,p,t – computational prediction by miRbase, PicTar, TargetScan

Table S1 contains the set of all previously observed target pairs used in this study. Alternative nomenclature for miRNAs / genes is provided. Targeted genes are labeled (C) or (TR) depending on whether the target pair’s annotation in TarBase indicates previously observed evidence of mRNA cleavage or translational repression respectively. The citation provided by TarBase justifying the targeting relationship is also provided.
