# Supplementary material for: Statistical Use of Argonaute Expression and RISC Assembly in microRNA Target Identification
Source: PLoS Comput Biol. 2009 Sep 25;5(9):e1000516. doi: 10.1371/journal.pcbi.1000516 (PMC2739424; doi:10.1371/journal.pcbi.1000516)
Supplement: Table S2 — Predicted m/miRNA target pairs. Table S2 contains the set of all predicted target pairs analyzed in this study. For each miRNA Table S2 provides the numbers of individual and simultaneous predictions made by miRBase and TargetScan for which the Madison dataset measured above noise expressions, along with a list of the genes analyzed. Predictions verified by use of model (2.3) are highlighted. (0.04 MB DOC) [file pcbi.1000516.s003.doc]

**Table S2. Predicted m/miRNA target pairs.**

| **miRNA** | **TargetScan / miRBase / Simultaneously predicted targets** | **Target List** |
| --- | --- | --- |
| miR-130b | 319 / 428 / 45 | ABCC5, ACBD5, ARHGAP21, CAMTA1, CD69, CHD9, DDX6, DLL1, DYNLL2, EDA, ELK3, FASTK, GADD45A, GAP43, GRLF1, MAP3K12, MIER1, MLL, MLLT10, MLLT6, MPHOSPH9, MTF1, NDEL1, NME7, PFKFB3, PLCB1, PPARG, PSAP, PTPN2, RAB34, RNF38, SNX2, SNX5, SPG20, SPHK2, STARD13, STIM2, TES, TNRC6A, TPP1, TRIM3, UCP3, WDR47, YTHDF2, ZNF3 |
| miR-133a | 225 / 324 / 36 | ANGPTL4, ATP6AP2, CCNC, CLTA, CSF2, CSNK1G3, DNM2, DUSP1, EIF4A1, ELF2, EVI1, FOXC1, GDI2, KIAA0241, KIAA1429, MRC2, MYCBP2, NDRG1, PCGF2, PPP2CB, PTHR1, PTPRD, RAG1AP1, RAPH1, RBMX, RCE1, RING1, SEC61B, SMARCD1, SNX1, SQLE, TAGLN2, TFAP2D, VPS54, ZNF131, ZNF385 |
| miR-133b | 225 / 357 / 36 | ANGPTL4, ATP6AP2, BRUNOL4, CCNC, CLTA, CSF2, CSNK1G3, DNM2, DUSP1, ELF2, EVI1, FOXC1, GDI2, KIAA0241, KIAA1429, MRC2, MYCBP2, MYH9, NDRG1, PPP2CB, PTHR1, PTPRD, RAG1AP1, RAPH1, RBMX, RCE1, RING1, SEC61B, SMARCD1, SNX1, SQLE, TAGLN2, TFAP2D, VPS54, ZNF131, ZNF385 |
| miR-15a | 451 / 397 / 77 | ANKRD15, AP2A1, ATXN2, CAPZA2, CARD10, CASZ1, CCNT2, CDCA4, CHEK1, CHPT1, COPS2, CRKL, CUL2, DLL1, DMTF1, EDA, ENAH, FAM91A1, GLUD2, HDGF, HIGD1A, HTR4, IGF1R, IL1RAPL1, INSR, KBTBD2, KIAA0241, KIF21A, KLC4, LRP6, MAP2K1, MYB, NFATC3, NKD1, NRP2, ODZ2, OTUB1, PAK7, PCDH9, PCMT1, PDLIM5, PIAS1, PLEKHC1, PPAP2A, RAD9A, RANBP3, RASGEF1B, RBBP6, RBM6, RSBN1, SCOC, SEMA6D, SERBP1, SESN1, SFRS11, SFRS16, SIAH1, SIDT2, SNTB2, SRPK1, SRPR, STK19, STOX2, STXBP1, STXBP3, TAF15, TBPL1, TFAP2D, TLE4, TSPAN5, VAMP8, WBP11, WEE1, WIBG, YWHAQ, ZNF622, ZYX |
| miR-16 | 451 / 381 / 76 | ADAMTS18, AP2A1, ATXN2, CARD10, CASZ1, CCNT2, CHEK1, CHPT1, CHRNE, COPS2, CRKL, CUL2, DIXDC1, DLL4, EDA, ESRRA, FAM91A1, FGFR1, GLUD2, GORASP2, HDGF, HIGD1A, HSPA4L, HTR4, ISOC1, KCNAB1, KIAA0241, KIF21A, KLC4, LPHN2, LRP6, LYPLA2, MYB, NFATC3, NKD1, ODZ2, OTUB1, PCBP4, PCMT1, PIAS1, PLEKHC1, PPAP2A, PRPF38A, RAD9A, RANBP3, RASGEF1B, RBM6, RET, RSBN1, SCOC, SEMA6D, SERBP1, SESN1, SFRS11, SFRS16, SH3GL2, SIAH1, SNTB2, STK19, STOX2, STXBP3, TAF15, TFAP2D, TGFBR3, TLE4, TSPAN5, VAMP8, VAV2, WBP11, WEE1, WIBG, YTHDC1, YWHAQ, ZBTB10, ZNF622, ZYX |
| miR-181a | 463 / 433 / 66 | ACSL1, ADAMTS18, ARNT2, ATG5, ATP11C, BAI3, BCL11A,  BRD1, BRWD1, C1orf149, CAMSAP1, CARD11, CD163, DOCK10, DOCK7, EED, EIF4A2, FKBP1A, FOS, FOXP1, GHITM, GOLGA8A, GPBP1, HCN2, HMGB2, ITGB8, KCNMA1, KIAA0195, KIAA0528, KIAA0802, KLF6, LMBRD2, LRBA, MYBL1, MYCBP2, NIPBL, NMT2, NPTN, NR6A1, NSMAF, PAM, PAWR, PCGF2, PDIA6, PHLDA1, PLCL2, PRKAG2, PROX1, PSPC1, RALA, RBBP7, RBM22, RGMA, RNF34, RNF6, SACM1L, SEMA4C, SIPA1L2, SLMAP, TBPL1, TCERG1, TGFBI, TNFRSF11B, TOM1L1, TRIM3, ZNF207 |
| miR-181b | 463 / 448 / 67 | ADM, ANKRD15, ATG5, ATP2A2, BAI3, BRWD1, C10orf22, CARD11, CD163, CNNM2, DAZAP2, DMXL2, DOCK10, DOCK7, EIF4A2, FKBP1A, FOXP1, GOLGA8A, GPBP1, HCN2, HMGB2, KCNMA1, KIAA0195, KIAA0802,  KLF6, KLHL5, KPNA1, LEMD3, LMBRD2, MYO1C, NIPBL, NPTN, NR6A1, NSMAF, PAM, PAWR, PCAF, PCGF2, PCNP, PDIA6, PKNOX2, PLCL2, PPP1R12B, PRKAG2, PROX1, PSPC1, RNF34, RNF6, RTF1, SACM1L, SEMA4C, SEMA4G, SFRS7, SIPA1L2, SLC25A37, SLMAP, STAG1, TARDBP, TBL1XR1, TBPL1, TGFBI, TNPO1, TOM1L1, TRIM3, ZC3H11A, ZDHHC7, ZNF207 |
| miR-181c | 463 / 444 / 68 | ABI1, ACSL1, ANKRD15, BAI3, BCL11A, BRD1, CARD11,  CD163, CD4, CNNM2, DEK, DMXL2, DOCK10, DOCK7,  EIF4A2, FKBP1A, FNDC3A, FOS, GDPD1, GOLGA8A, GPBP1,  HCN2, HMGB2, ITGB8, KCNMA1, KIAA0195, KIAA0528, KIAA0664, KIAA0802, KLF6, KLHL5, KPNB1, LMBRD2, MYBL1, NIPBL, NMT2, NR6A1, NSMAF, PAWR, PBX3, PCGF2, PDIA6, PHLDA1, PIK3R3, PLCL2, PLEKHA3, PRKAG2, PROX1, PRTG, PSPC1, RAD21, RAN, RBBP7, RBM22, RGMA, RNF34, SEMA4C, SIPA1L2, SLMAP, TBPL1, TCERG1, TGFBI, TNFRSF11B, TOM1L1, TRIM3, TSC22D2, WSB1, ZNF207 |
| miR-192 | 63 / 315 / 17 | ARFGEF1, ATF1, BLCAP, CCNT2, COL5A1, DBT, DYRK3, LARP4, LPHN3, NIPBL, PABPC4, PKP4, PPP1CA, RABGAP1, RB1, TCF7, ZBTB38 |
| miR-211 | 200 / 257 / 18 | AKAP1, ATF2, AUP1, C21orf63, CCNT2, CHD5, CNTNAP2, COX5A, DNM2, EEF1E1, FRAS1, IL23A, KCNMA1, LRRC8D, SASH1, SEC24D, SSRP1, TRIP12 |
| miR-212 | 178 / 406 / 35 | ACVR2B, AFF4, ARMC1, AZIN1, BNC2, BRI3, BTG2, CALU, CBX1, DEDD, DYNLL2, EIF4A2, FOXO3A, FOXP1, H2AFZ, HBEGF, HNRPM, HNRPU, JARID1A, LRRFIP1, MYCBP2, NFIB, PAIP2, PEA15, PNN, POM121, PPM1G, PRPF4B, RASA1, SEMA4G, TAF15, USP9X, VDAC2, ZCCHC11, ZNF644 |
| miR-217 | 135 / 336 / 27 | ANLN, BAI3, C14orf37, CHN2, CUL5, DKK1, EIF4A2, EZH2, FBN2, FBXO11, GRIK2, HNRPUL1, LIN9, NOVA1, NPTN, PCNA, POLG, SLC38A2, STAG2, STRBP, TACC2, TLK2, UBL3, USP15, VSNL1, YTHDC1, ZFYVE20 |
| miR-224 | 144 / 371 / 30 | AP2M1, ARHGAP21, ATF2, ATP1B3, CAST, COL7A1, DNM1, DPYSL2, FAM38B, FAM49B, FTH1, HES5, ITM2B, KCNMA1, KIF5C, MGAT4B, NCOA6, NUP153, QKI, RAD51L1, RNF38, SPEN, STMN1, TCERG1, TMEM117, TMEM9B, XAB1, ZDHHC20, ZNF207, ZNF403 |
| miR-29a | 399 / 411 / 84 | ABCB6, AIM1L, ANKRD49, ARVCF, ATP5G1, BACH2, BLMH, C11orf30, C19orf6, CALU, CAMTA1, CASZ1, CCNL2, CDC42, CDCA4, CLK2, COL11A1, COL1A2, COL3A1, COL4A1, COL4A5, COL6A3, COL7A1, COMMD2, CSDA, DIABLO, DNAJB11, DPF1, DPYSL2, EIF4E2, ELN, FBN1,  FOS, GAB1, GPR37, HAS3, HBP1, HIF3A, HMGCR, HMGN3,  HNRPUL1, IFI30, IREB2, JMJD3, KLF4, KLHDC5, LARP5, MGAT4B, MLF1, MYCN, NASP, NCOR2, NFIA, NUP160, PAIP2, PARG, PDGFC, PDHX, PMP22, POU2F2, PPIC, PRKRA, REV3L, SESTD1, SETDB1, SMS, SPTAN1, SRCRB4D, SUV420H2, TDG, TFEB, TNFRSF1A, TPM1, TRAF4, TRIM37, TSPAN14, TSPAN4, USP34, VPS25, XKR6,  ZBTB5, ZDHHC5, ZFP36L1, ZFP91 |
| miR-29c | 399 / 405 / 85 | ABCB6, ADAMTS18, AIM1L, ANKRD49, ARVCF, ATP5G1, BACH2, BLMH, C11orf30, C19orf6, C5orf13, CAMTA1, CASZ1, CCNL2, CLK2, COL11A1, COL1A2, COL3A1, COL4A1, COL4A5, COL6A3, COL7A1, COMMD2, CSDA,  DIABLO, DNAJB11, DPF1, DPYSL2, EIF4E2, ELF2, FBN1, FOS, GAB1, GPR37, HBP1, HIF3A, HMGCR, HMGN3, HNRPUL1, IFI30, IREB2, JMJD3, KLF4, LARP5, MGAT4B, MLF1, MYCN, NASP, NCOR2, NFIA, NUP160, OTUD4, PAIP2, PARG, PDGFC, PDHX, PLEKHC1, PMP22, PPIC, PRKRA, REV3L, RND3, SESTD1, SETDB1, SGK, SMS, SPEN, SPTAN1, SRCRB4D, SUV420H2, TDG, TFAP2C, TFEB, TNFRSF1A, TPM1, TRAF4, TSPAN14, TSPAN4, USP34, VPS25, XKR6, ZBTB5, ZDHHC5, ZFP36L1, ZFP91 |
| miR-30d | 552 / 409 / 89 | ANKRA2, AP2A1, AP3S1, ARID1A, ARL6IP6, ATP2A2, BCL9, BCOR, BECN1, C13orf18, C9orf86, CACNB2, CADPS, CAMK2N1, COL13A1, CPSF6, CSDA, CUL2, DCX, DDIT4, DLG5, DLL4, DOCK7, EED, EPHB2, ESCO1, FAM43A, FBXL20, FBXO34, FGD6, FKBP3, FRMD6, GALNT2, GLCCI1, GLDC, GLI2, GNAO1, GRK5, GRM5, HERC2, IRS1, JAG2, JMJD1A, KCNJ3, KCTD5, KIAA0241, KSR1, LRFN2, LRRC8D, MAP3K12, MAP3K5, MICAL1, MOV10, NCOR2, NEUROD6, NFIB, NHLH2, OTUD4, P4HA2, PAWR, PCDH19, PGM1, PIK3CD, PNN, PON2, PPARGC1B, PPID, PRLR, PTPN13, RAB32, RAB38, RGS2, RRAD, SAP30, SBF1, SEMA6D, SLC5A11, SOCS1, SPEN, STOX2, TIA1, TMEFF1, TNRC6A, TNXB, UBE2I, USP48, YTHDC1, ZNF644, ZNRF1 |
| miR-326 | 136 / 295 / 12 | ABCC1, ARID5B, AUP1, C9orf24, DRD2, EHMT2, EML2, ESRRA, GPI, HMGA2, NR6A1, TSPAN14 |
| miR-34b | 155 / 406 / 6 | ARID1B, BRD4, C21orf66, FAM49B, FOXP1, PPP3CA |

Table S2 contains the set of all predicted target pairs analyzed in this study. For each miRNA Table S2 provides the numbers of individual and simultaneous predictions made by miRBase and TargetScan for which the Madison dataset measured above noise expressions, along with a list of the genes analyzed. Predictions verified by use of model (2.3) are highlighted.
